# Examining the Causes and Consequences of Short-Term Behavioral Change during the Middle Stone Age at Sibudu, South Africa

**DOI:** 10.1371/journal.pone.0130001

**Published:** 2015-06-22

**Authors:** Nicholas J. Conard, Manuel Will

**Affiliations:** 1 Department of Early Prehistory and Quaternary Ecology, University of Tübingen, Schloss Hohentübingen, 72070, Tübingen, Germany; 2 Senckenberg Center for Human Evolution and Paleoecology, University of Tübingen, Schloss Hohentübingen, 72070, Tübingen, Germany; University of Oxford, UNITED KINGDOM

## Abstract

Sibudu in KwaZulu-Natal (South Africa) with its rich and high-resolution archaeological sequence provides an ideal case study to examine the causes and consequences of short-term variation in the behavior of modern humans during the Middle Stone Age (MSA). We present the results from a technological analysis of 11 stratified lithic assemblages which overlie the Howiesons Poort deposits and all date to ~58 ka. Based on technological and typological attributes, we conducted inter-assemblage comparisons to characterize the nature and tempo of cultural change in successive occupations. This work identified considerable short-term variation with clear temporal trends throughout the sequence, demonstrating that knappers at Sibudu varied their technology over short time spans. The lithic assemblages can be grouped into three cohesive units which differ from each other in the procurement of raw materials, the frequency in the methods of core reduction, the kind of blanks produced, and in the nature of tools the inhabitants of Sibudu made and used. These groups of assemblages represent different strategies of lithic technology, which build upon each other in a gradual, cumulative manner. We also identify a clear pattern of development toward what we have previously defined as the Sibudan cultural taxonomic unit. Contextualizing these results on larger geographical scales shows that the later phase of the MSA during MIS 3 in KwaZulu-Natal and southern Africa is one of dynamic cultural change rather than of stasis or stagnation as has at times been claimed. In combination with environmental, subsistence and contextual information, our high-resolution data on lithic technology suggest that short-term behavioral variability at Sibudu can be best explained by changes in technological organization and socio-economic dynamics instead of environmental forcing.

## Introduction

Researchers studying the cultural evolution and Paleolithic lifeways of hominins conduct their work at multiple scales of analysis, including temporal, spatial, demographic and behavioral dimensions [1–3]. Archaeologists can consider actions of hominins on the scales of seconds or many hundreds of thousands of years. Spatial scales can range from microscopic to continental. When examining the demographic dimension, the individual is the smallest unit of analysis, while studies can also address the population dynamics of entire species. Finally, the behavior scale spans the range of hominin experience from the fulfillment of essential biological needs for food, drink and sleep to a wide range of practical activities as well as abstract thoughts and beliefs.

Studies of the Middle Stone Age (MSA) and Middle Paleolithic, which span the period from roughly 300 ka to 30 ka, have addressed all these scales with mixed results. Each area of research and multiple schools of thought approach these issues from different perspectives with specific goals and methods [2, 4–8]. For example, Middle Paleolithic research in Europe has often addressed scales of high spatial and temporal resolution at the level of individuals and small social units. Work at sites including Maastricht-Belvédère [9], Tönchesberg [10, 11], Wallertheim [12, 13], and Abric Romani (articles in [8, 14]) reflect examples of high-resolution archaeology, often based on refitting studies of lithic artifacts and faunal material. Such approaches have generally not been the emphasis of researchers working in the MSA, with few notable exceptions such as Van Peer *et al*.*´*s [15] work at Taramsa I in Egypt, although Wadley [16, 17] has advocated similar strategies.

Here we aim to examine the high-resolution cultural stratigraphic sequence of Sibudu in KwaZulu-Natal, South Africa, to consider the nature and potential explanations for short-term changes in behavior during the MSA. In the case study presented here, we are less concerned with the paleo-ethnographic scale (e.g. [18–20]) which focuses on the actions of individuals and small social units over periods of seconds, hours, days, months or years and their implications for the material record of the past. Instead, we consider how the rich archaeological record from Sibudu preserves a record of short-term cultural change over what likely spans the temporal scale of individual lives, decades and centuries.

Based on the available OSL dates, the eleven archaeological strata from Sibudu that we analyze here are indistinguishable in age and date to around 58,000 years ago (= 58 ka), ca. 4,000 years younger than the underlying Howiesons Poort (HP) occupations [21, 22]. The focus of this paper is to examine how we can use these high-resolution signatures of changing lithic assemblages to gain new insights into the nature and potential causes of changes in behavior and technology in a sequence of multiple find horizons that all fall within the uncertainty of the resolution of radiometric dating. Generally research in the MSA has not addressed this scale of variation, although previous research at Sibudu (e.g. [17]) or Diepkloof [23, 24] used similar approaches. Here we look at short-term technological change during the early phase of MIS 3 to illuminate potential factors that shape cultural evolution. These observations have implications for ongoing debates about the rates and causes of cultural change during the MSA and the reasons why some innovations come and go, while others spread and shape technological systems in the long-term [22, 23, 25–30].

More specifically, we examine 11 find horizons in the period that we elsewhere have defined as the Sibudan [31, 32]. While the upper part of the sequence, find horizons BM-BSP, reflects the type assemblages for this cultural entity, we here report different technological signature for the lower stratigraphic units. This variability raises fundamental questions about the causes and scope of technological changes and begs the question of how much variation can be included in any cultural taxonomic unit. Following J. Brew [33], we are very much aware that there are no universally valid answers to these questions, since any taxonomy has meaning only within the contexts of specific questions researchers pose. Nonetheless, in the case of the Sibudan and the cultural stratigraphy of the MSA, researchers need to be explicit about these questions, since often we have simply continued to use the cultural taxa defined by Goodwin and van Riet Lowe [34] over 85 years ago based on the poor data that were available at that time.

We also consider what approaches will likely provide insight into the causes and implications of cultural change during early MIS 3. We draw on organization of technology of and evolutionary approaches to cultural change to see if they can help us assess why innovations occur, persist or disappear and to examine what selective pressures shape MSA technology. More specifically, we investigate whether changes in demography, environment, subsistence and other socio-cultural dynamics were causal mechanisms of behavioral change at Sibudu.

## Materials and Methods

### Sibudu and its high-resolution MIS 3 deposits

**Fig 1:**
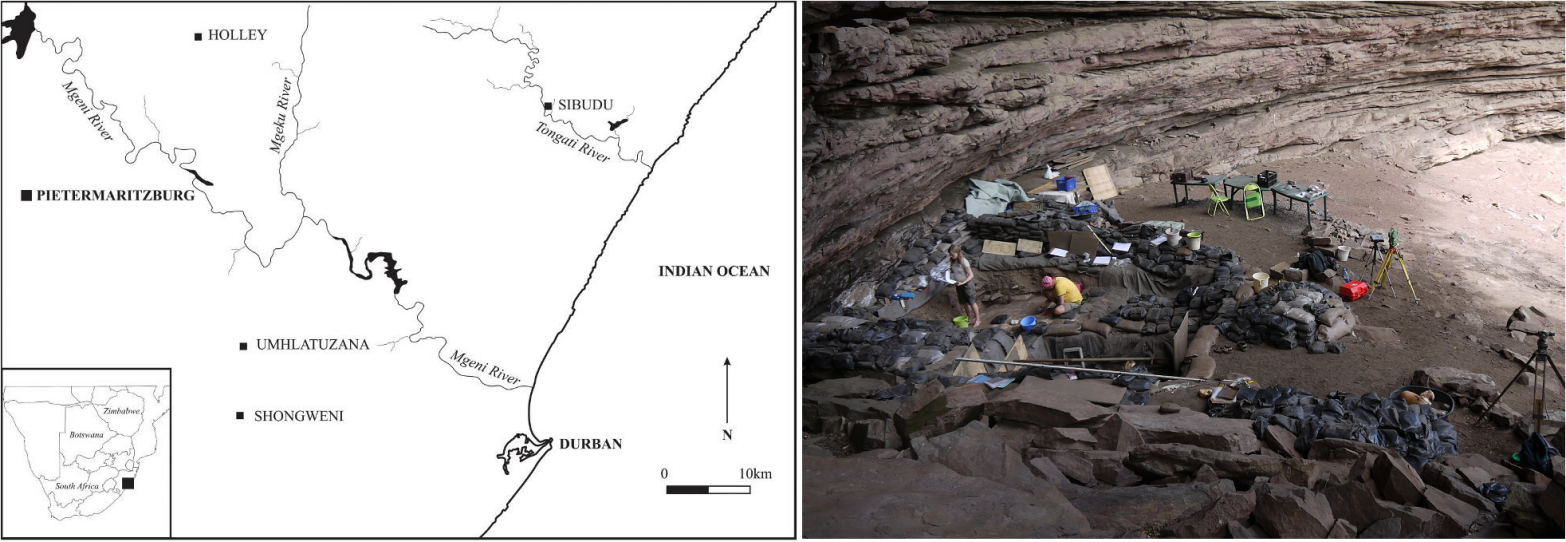
Geographical location of Sibudu in KwaZulu-Natal and view on the excavation area within the rock shelter.

Sibudu is a large rock shelter located above the Tongati River (also spelled "uThongathi") about 40 km north of Durban and 15 km from the Indian Ocean in the KwaZulu-Natal region (Fig 1). The site hosts a rich and thick archaeological sequence with deposits that have been dated to >77–37 ka, preserving more than 40,000 years of MSA occupations. In terms of cultural chronology, Sibudu has yielded evidence for Still Bay (SB) and Howiesons Poort (HP) occupations, but also for the periods before and after. The record from MIS 3 is particularly rich, consisting of pulses of occupation at ~58 ka, ~48 ka and ~38 ka [21, 22, 31, 35].

The stratigraphic framework of the site results from long-term excavations by L. Wadley that began in 1998 and continued until 2011. The MSA sequence at Sibudu is over 3 meters thick and is characterized by largely anthropogenic sediments, features good organic preservation and little post-depositional disturbance [36–40]. The 11 lithic assemblages of this study derive from the upper portion of the ~1.5 meter thick sediments that overly the HP. This "post-HP" sequence at Sibudu contains over 20 archaeological horizons consisting of finely laminated strata [21, 32] (see also [35]: Fig 1). Occupations at the top and base of this thick sequence have been dated indistinguishably to ~58 ka by OSL, providing an exceptionally high temporal resolution for an MSA site [21, 22]. These layers likely accumulated over a period of centuries and constitute one of the best and thickest stratigraphic records of this period known anywhere. They present an ideal case study for analyzing high resolution MSA behavioral variability. Based on detailed lithic analyses, we recently proposed that the uppermost 6 layers of these deposits form part of the “Sibudan”, a new cultural taxonomic unit of the later MSA [31, 32].

The excavations by Wadley and our own team have documented very high densities of archaeological finds in these Sibudan strata. Additionally, both absolute dating and geoarchaeological analyses suggest high rates of anthropogenic sedimentation. The sequence exhibits multiple hearths, bedding and other indications of site use and maintenance. These observations document that modern humans made very intense use of the site around ~58 ka [21, 31, 32, 38–41].

### Methods of excavation

New field work at Sibudu has been carried out by a team of the University of Tübingen under the direction of N. Conard since 2011. The research permit to conduct archaeological excavations at Sibudu is issued under the KwaZulu-Natal heritage Act No. 4 (permit number: REF: 0011/14; 2031CA 070). All recovered archaeological specimens are housed in the KwaZulu-Natal Museum in Pietermaritzburg. The specimen numbers of this study are C2.6–1439; C3.2–1141; D2.2–1028; D3.1–1364; E2.4–1151; E3.2–1457 (including sub-numbers).

The current research team adopted Wadley´s stratigraphic system and designations (see [21] Table 2). The find horizons have been excavated with careful piece-plotting of artifacts, using a Leica total station and the EDM program [42], with great attention being paid to establish reliable high-resolution cultural chronological units. We do this by following the concept of excavating *Abträge* (singular *Abtrag*) that follow the contours of the stratigraphic sequence. In keeping with this approach, our excavations proceeded carefully in 1–3 cm thick *Abträge* in each quarter meter, following the slope of the sediments without crosscutting geological strata. We group these *Abträge* in larger units that we call find horizons. Given the rapid rate of sedimentation and the high occupation intensity at Sibudu, this excavation method allows us to examine patterns of change in the material culture of the site’s inhabitants in great detail. Due to this careful strategy, we are confident in assessing the provenience of each artifact and thus in the integrity of the lithic assemblages of this study. We recorded the volume of all excavated sediments and sieved every buckets of sediment through nested screens with 5 mm and 1 mm mesh in order to recover small finds.

### Materials and methods of lithic analysis

**Fig 2:**
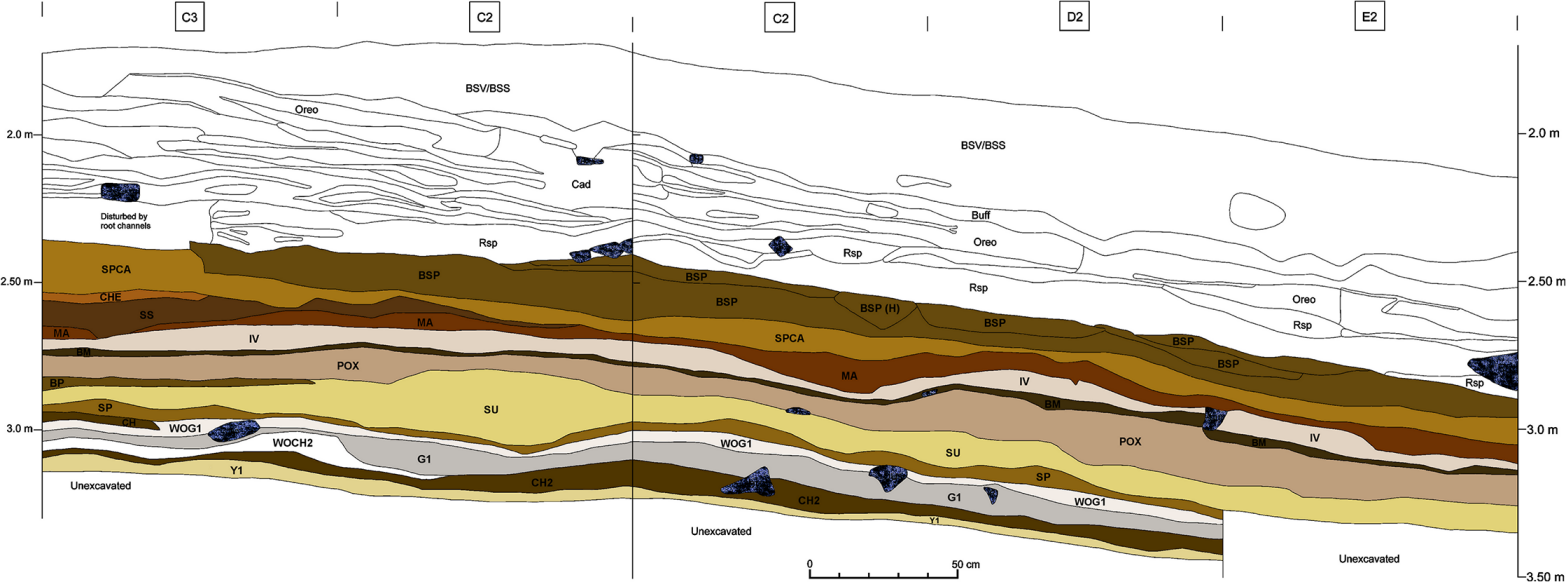
Stratigraphic section of the Eastern Excavation (combined north and east profile) of Sibudu. Colored layers, beginning with BSP, were excavated by the Tübingen team between 2011–2014 and are located in the upper part of the sequence dated to ~58 ka.

This study includes all lithic finds from layers WOG1-BSP from the 2011–2014 Tübingen excavations, which reflects an area of excavation of 6 m^2^ and a volume of excavation of about 3 m^3^ (Fig 2). We analyzed a total of eleven assemblages, with one layer (SS) being excluded due to the low number of artifacts (n<100). Due to the high density of lithic artefacts, we used 30 mm as the cut off for single finds. The eleven assemblages include a total of 146,301 stone artifacts, with 7,799 pieces >30 mm and 138,502 small debitage products <30 mm (Table 1). The large number of successive layers and lithic finds allows for an excellent assessment of diachronic variability throughout this part of the sequence. The very high ratio of small debitage to single finds (95:5%) is indicative of intense stone knapping with little post-depositional disturbance or sorting based on size (S1 Fig).

**Table 1:** Distribution of lithic single finds (>30 mm) and small debitage (<30 mm).

Layer	Single finds	Small debitage	Total lithics
BSP	822	13644	14466
SPCA	578	10019	10597
CHE	133	2792	2925
MA	178	4421	4599
IV	676	20389	21065
BM	262	5694	5956
POX	2192	43418	45610
BP	261	4039	4300
SU	1624	26816	28440
SP	705	5060	5765
WOG1	368	2210	2578
Total	7799	138502	146301

The aim of this study is to document and interpret short-term behavioral change in a high-resolution sequence. In order to achieve this goal and maximize the amount of pertinent information, we follow an approach to lithic analysis that combines the virtues of several complementary methods:

- Reduction sequence analysis [12, 43–47] evaluates the methods of core reduction and the stages of knapping, use and discard of stone artifacts that people performed at the site. We include only lithic artifacts larger than 30 mm.
- Attribute analysis [48–53] informs on technological behaviors by providing quantitative data of the numerous discrete and metric traces on individual artefacts that result from the knapping process. As part of this method, techno-economic approaches measure reduction intensities [54–56], calculate flaking efficiencies [57, 58] and assess raw material economy [59–63]. Only stone artifacts >30 mm were included.
- Typological [64, 65] and techno-functional analyses [66–70] provide comparable data on tool types across sites and regions and study the technological approach of knappers towards producing, using and recycling tools.
- Quantifying small lithic artifacts (30–5 mm) according to raw material aids in calculating find densities and patterns in the raw material economy. Identifying retouch debitage quantifies the level of on-site tool production and recycling.

Combing these methods, we reconstruct technological strategies of the individual assemblages by providing information on procurement and use of raw materials, reduction sequences, techniques of blank production and the approach used for tool manufacture and maintenance.

In order to help quantify the amount of retouching conducted on-site for each assemblage, we analyzed a representative sample of small debitage products (<30mm) for retouch flakes. Retouch flakes among the small debitage were identified using the following characteristics: 1) a plain striking platform with a lip; 2) an obtuse angle between striking platform and ventral surface; 3) the existence of dorsal negatives that originate from the previously retouched edge; 4) an overall divergent fan-like morphology; 5) an abrupt, hinge or plunge termination (see [31]: Fig 14). We classified the piece as retouch debitage if at least three of five characteristics were present, yielding conservative estimates for on-site retouching.

Our analyses of core reduction methods follow the unified taxonomy by Conard *et al*. [71] in order to provide a classification which is applicable to all periods without temporal or spatial restrictions. In this taxonomy, “parallel”, “inclined” and “platform” constitute three main types of core reduction. Conard *et al*. ([71]: 15–16) define their core categories as follows. Parallel cores “have two surfaces whose main removal surface must include one or more major removals parallel to the plane that intersects the two surfaces […] These cores are usually asymmetrical in cross-section with a slightly convex main removal surface and a more inclined ‘underside’. All significant removals originate from the intersection of the two surfaces”. Inclined cores on the other hand “have two surfaces with removals inclined relative to the plane defined by the intersection of the surfaces. Either or both surfaces may be used for the main removals. The removals have an angle of roughly 45° relative to the plane of intersection. All significant removals originate from the intersection of the two surfaces”. Finally, platform cores “have more than two faces and are not defined by the plane of intersection of two surfaces as in the above two approaches. Removals do not need to be on the broad surface of the core and are often on narrow surfaces. One or more well organized and well developed striking platforms with three or more contiguous, successful removals from the corresponding knapping surfaces must be recognizable”. This category encompasses what is elsewhere referred to as single and multi-platform as well as so-called rotated cores for the production of flakes, blades or bladelets.

In order to overcome limitations of a typological approach [53, 72, 73], we also employed a techno-functional method for the analysis of retouched specimens which divides tools into a transformative, prehensile and intermediate part and studies the treatment of these portions separately [66–70]. In combination with this concept, we also focused on the reduction and transformation of tools (e.g. [74–77]), emphasizing their dynamic nature instead of considering only their final state of modification and discard. Using these methods, we previously classified tools based on the identification of specific patterns of repetitive retouch on different parts of the tool which indicate formal and distinct retouching sequences [31, 32]. The four main techno-functional tool classes and reduction sequences at Sibudu comprise Tongatis, Ndwedwes, naturally backed tools (NBTs), and asymmetric convergent tools (ACTs). Although these tool classes do not function as type fossils, their frequent and joint occurrence is part of the original definition of the “Sibudan” [31].

The hallmark of Tongati tools is their short triangular distal end, which is usually retouched in a symmetric manner on both working edges of the point. Tongatis are continuously reduced from the distal to the proximal end–becoming shorter as retouch progresses–but they always retain their convergent distal configuration (see [31]: Fig 5–8). ACTs are similar to Tongatis, but the distal tip is always asymmetrical. Most specimens have steeper, retouched edges opposed to a sharp non- or only marginally retouched edge (see [32]: Fig 5). ACTs typically change at their initially unretouched working edge, as use-wear and edge damage accumulate, decreasing the width of the piece during their use life. “Ndwedwe” tools comprise retouched forms that are elongated, thick pieces with modifications along the lateral edges. They are characterized by their steep and invasive lateral retouch that usually runs the entire length of both sides of the tool. In contrast to Tongati tools, Ndwedwes begin with relatively broad forms and become narrower and narrower with progressive retouch, while the length remains nearly constant over the course of reduction (see [31]: Fig 11). Finally, NBTs are characterized by a natural back–including Siret fractures, other kinds of breaks and cortical edges–opposite to the retouched edge of the piece (see [31]: Fig 12). Due to the existence of a thick back, NBTs usually possess an asymmetric cross-section. More detailed descriptions, discussion of their function and additional drawings of these tool concepts and reduction sequences can be found in Conard *et al*. [31] and Will *et al*. [32].

## Results

### Procurement and use of lithic raw materials

Knappers at Sibudu procured both local and non-local (>10 km distant) lithic raw materials. Local materials include dolerite, quartz, quartzite and sandstone, with non-local variants represented by hornfels, jasper and crypto-crystalline silicates (CCS). The local materials occur either directly at the shelter and its surroundings (dolerite, sandstone) or as pebbles in the Tongati River (quartz, quartzite). There are no known outcrops of hornfels, jasper and CCS in the direct area (10 km radius) around Sibudu. While their precise origins remain to be determined, the inhabitants of the site had to import these raw materials from some distance to the site [78, 79].

Accessibility, form of occurrence and knapping quality of raw materials, as well as social parameters influenced the choice of raw materials used by MSA hunter gatherers. From the point of view of fracture mechanics [80, 81], hornfels, CCS and jasper constitute the best materials at Sibudu. While these raw materials provide sharp edges, they are often fragile and prone to break (e.g. [79]). The local dolerite from Sibudu is a homogenous but hard and rough raw material. Knapping of dolerite requires considerable force and skill, but yields durable edges which do not break as easily as those of hornfels [79]. Quartzite and sandstone share similar qualities, with the latter being very coarse-grained. Due to its internal structure, quartz tends to break along crystal boundaries and not conchoidally but provides extremely sharp and durable edges [82–84].

**Fig 3:**
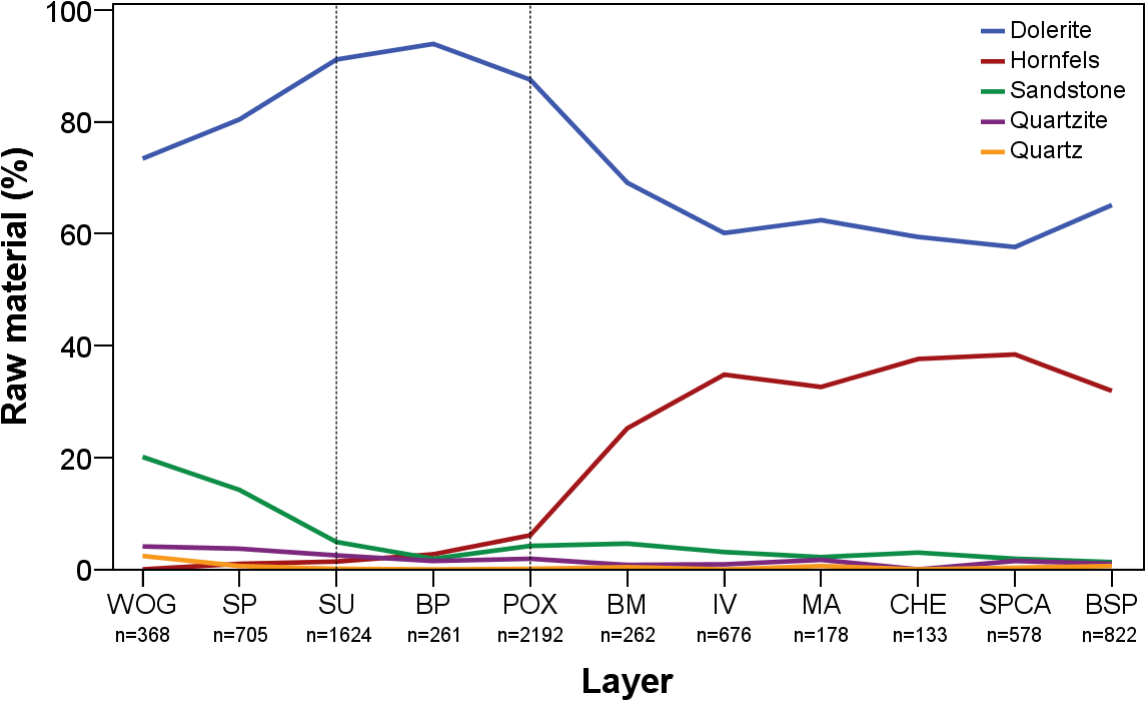
Percentual abundance of raw materials throughout WOG1-BSP. WOG1 = oldest layer; BSP = youngest layer.

Throughout the studied sequence, knappers gradually changed their selection of raw materials (Fig 3; Table 2). Dolerite constitutes the dominant tool stone in all layers, but varies in abundance. The two lowest assemblages WOG1 and SP yield values between 75–80%, followed by an almost exclusive use of dolerite in SU-POX (90–95%) and finally a drop in frequency to between 60–70% in the uppermost layers BM-BSP. In contrast to dolerite, we found unidirectional temporal changes for the selection of non-local hornfels and local sandstone. Hornfels is absent in the lowest assemblage WOG1 and increases gradually from SP (1%) to POX (6%). Layer BM marks a distinct break in the sequence with hornfels shooting up to 25%, reaching almost 40% in CHE and SPCA (Fig 3). From SU-BSP, decreases in dolerite correlate strongly with increases in hornfels (*r* = 0.87; *p* = 0.002). While sandstone is well-represented in the lower assemblages WOG1 and SP (14–20%), layers SU and above demonstrate only <5% of this raw material. Other local (quartz, quartzite) and non-local raw materials (jasper, CCS) played a negligible role for the inhabitants. In terms of raw material origins, all assemblages from the base of the studied sequence up to POX yield less than 6% raw materials from non-local sources–or none at all–whereas the upper layers BM-BSP exhibit a four- to six-fold increase in tool stones from further away to between 25–38%.

**Table 2:** Distribution of raw materials (>30 mm).

Layer	Dolerite	Hornfels	Sandstone	Quartzite	Quartz	Jasper	CCS/Other	TOTAL
BSP	535 (65%)	262 (32%)	11 (1%)	8 (1%)	5 (1%)	-	1 (0%)	822
SPCA	333 (58%)	222 (38%)	11 (2%)	9 (2%)	2 (0%)	-	1 (0%)	578
CHE	79 (59%)	50 (38%)	4 (3%)	-	-	-	-	133
MA	111 (62%)	58 (33%)	4 (2%)	3 (2%)	1 (1%)	1 (0%)	-	178
IV	406 (60%)	235 (35%)	21 (3%)	6 (1%)	-	8 (1%)	-	676
BM	181 (69%)	66 (25%)	12 (5%)	2 (1%)	1 (0%)	-	-	262
POX	1919 (88%)	134 (6%)	93 (4%)	42 (2%)	1 (0%)	-	3 (0%)	2192
BP	245 (94%)	7 (3%)	5 (2%)	4 (1%)	-	-	-	261
SU	1479 (91%)	22 (1%)	80 (5%)	41 (3%)	1 (0%)	-	1 (0%)	1624
SP	567 (80%)	7 (1%)	100 (14%)	26 (4%)	4 (1%)	-	1 (0%)	705
WOG1	270 (74%)	-	74 (20%)	15 (4%)	9 (2%)	-	-	368
Total	6125 (79%)	1063 (14%)	415 (5%)	156 (2%)	24 (0%)	9 (0%)	7 (0%)	7799

Rounded percentages are given in brackets.

In conclusion, knappers pursued three different strategies of raw material procurement (Fig 3). The lowest layers WOG1 and SP are characterized by the near absence of non-local raw materials, with the strongest focus on sandstone. Throughout SU-POX, the inhabitants of Sibudu almost exclusively used the local dolerite, with a small but gradually increasing amount of non-local hornfels. The upper layers BM-BSP exhibit an emphasis on the procurement of non-local hornfels associated with a decrease in dolerite and little use of other raw materials.

### Technological and techno-economic behavior

#### Analysis of debitage

**Fig 4:**
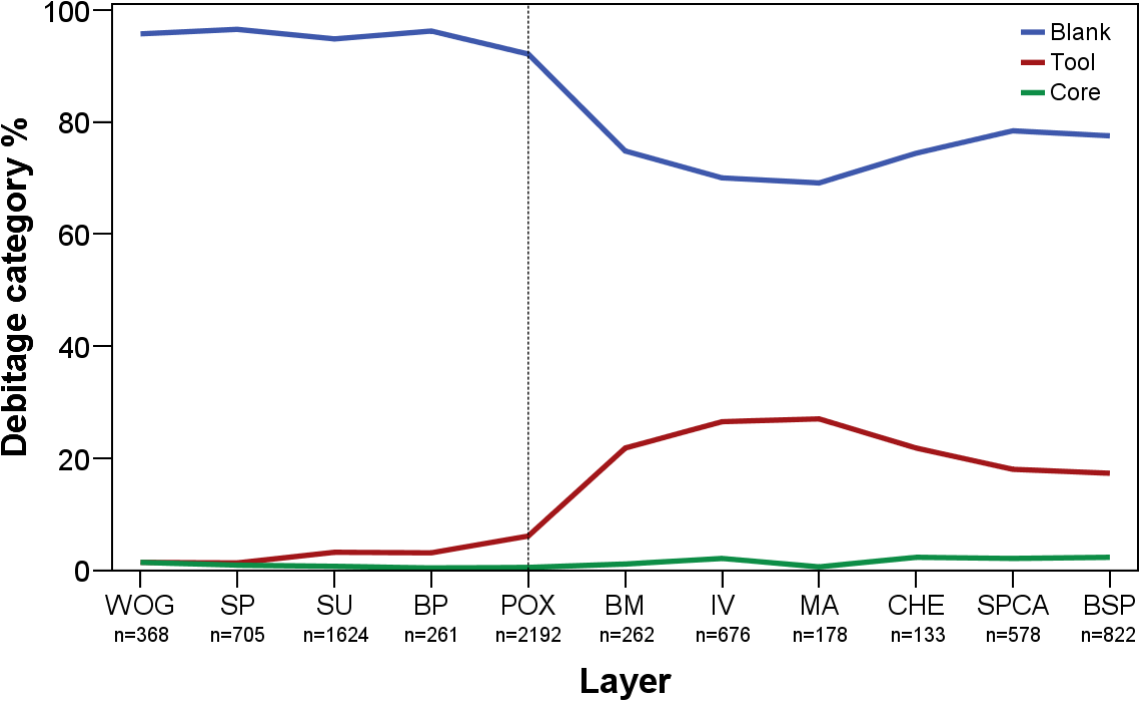
Frequencies of the main debitage category produced throughout WOG1-BSP. WOG1 = oldest layer; BSP = youngest layer.

The quantitative analysis of debitage for assemblages WOG1-BSP demonstrates marked diachronic changes (Table 3). Tools increase gradually throughout the sequence, with the transition from POX (6%) to BM (22%) being the most pronounced break (Fig 4). In general, WOG1-POX yield only few retouched specimens (1–6%), a standard value for most MSA assemblages (e.g. [85–87]). These figures stand in marked contrast to the upper assemblages BM-BSP for which the retouched lithic component is exceptionally high (17–27%). Not surprisingly, the number of unretouched blanks covaries with the frequency of tools. Cores and angular debris remain at low values throughout the studied sequence. The paucity of cores (n = 57; 1–2%) suggests that knappers reduced their raw materials intensely on-site and often exported non-exhausted cores.

**Table 3:** Quantitative debitage analyses of the main lithic categories (>30 mm).

Layer	Blank	Tool	Core	Angular debris	TOTAL
BSP	637 (78%)	142 (17%)	19 (2%)	24 (3%)	822
SPCA	453 (78%)	104 (18%)	12 (2%)	9 (2%)	578
CHE	99 (74%)	29 (22%)	3 (2%)	2 (2%)	133
MA	123 (69%)	48 (27%)	1 (1%)	6 (3%)	178
IV	473 (70%)	179 (26%)	14 (2%)	10 (2%)	676
BM	196 (75%)	57 (22%)	3 (1%)	6 (2%)	262
POX	2018 (92%)	133 (6%)	12 (1%)	29 (1%)	2192
BP	251 (96%)	8 (3%)	1 (0.5%)	1 (0.5%)	261
SU	1539 (95%)	52 (3%)	11 (1%)	22 (1%)	1624
SP	680 (97%)	9 (1%)	6 (1%)	10 (1%)	705
WOG1	352 (96%)	5 (1%)	5 (1%)	6 (2%)	368
Total	6821 (87%)	766 (10%)	87 (1%)	125 (2%)	7799

Rounded percentages are given in brackets.

For each assemblage, we analyzed retouch flakes in a representative sample of small debitage products (<30mm) to quantify the amount of on-site retouching. Based on the proportions of small retouch flakes in WOG1-BSP (total small debitage n = 22686; total retouch debitage n = 1645; S1 Table), the frequency of tools appears to be associated with their on-site production and curation. Just as in the overall tool proportions, there is a gradual increase of retouch flakes in the lowest layers WOG1-POX (~1–4%), followed by a distinct break from assemblage BM onwards (~11–24%). A statistical test of correlation confirms the strong co-variation between the proportion of retouch flakes (<30mm) and tool frequencies (>30mm) in the assemblages (*r* = 0.869; *p* = 0.001).

#### Production of blanks

**Fig 5:**
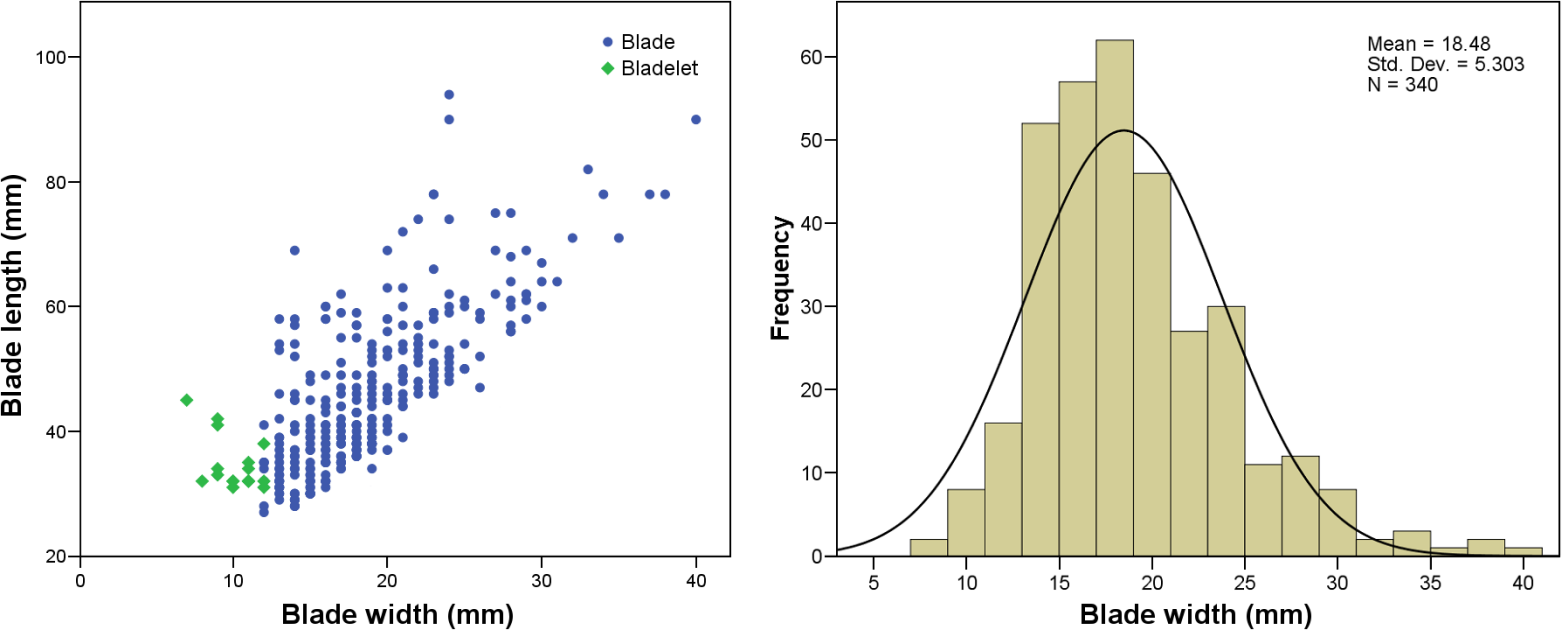
Scatter plot of blade widths and lengths (left) and histogram of blade widths (right) for the combined sequence WOG1-BSP.

Knappers at Sibudu produced a variety of types of blanks including flakes, convergent flakes, and blades, as documented from the blanks themselves and the cores used to produce them (Table 4). Flakes of various morphologies and sizes make up the majority of blanks in all layers. Although in lower numbers, the manufacture of convergent flakes and blades constitutes important elements throughout the sequence, an observation backed by frequent retouching of these blank types (see below). The only temporal difference in blank manufacture concerns the lowest layers WOG1 and SP. Not only is the blade component by far the lowest (5%), but many of these specimens also appear to be by-products. Hence, it is unclear whether an independent strategy of blade production exists in these assemblages. Although there are cores for the production of bladelets (n = 11), we found little evidence for the products them**s**elves. A scatter plot of length and widths of all laminar products and a histogram of widths only (Fig 5) support this observation. Both diagrams exhibit normal distributions around one peak, with the recovered bladelets rather as by-products of the continuous reduction of blade cores. We will further investigate whether or not this observation is an artifact of the size cut-off point of 30 mm used in this study.]

**Table 4:** Distribution of blank types^1^ (>30 mm).

Layer	Flake	Convergent flake	Blade	Bladelet
BSP	552 (71%)	88 (11%)	136 (18%)	2 (0%)
SPCA	418 (76%)	69 (12%)	68 (12%)	-
CHE	102 (80%)	12 (9%)	14 (11%)	-
MA	130 (76%)	22 (13%)	19 (11%)	-
IV	435 (67%)	105 (16%)	106 (16%)	5 (1%)
BM	165 (65%)	36 (14%)	50 (20%)	2 (1%)
POX	1591 (74%)	203 (9%)	185 (16%)	14 (1%)
BP	187 (72%)	40 (16%)	29 (11%)	3 (1%)
SU	1198 (75%)	193 (12%)	185 (12%)	14 (1%)
SP	569 (83%)	83 (12%)	36 (5%)	1 (0%)
WOG1	294 (83%)	43 (12%)	18 (5%)	-
Total^1^	5640 (74%)	894 (12%)	1001 (13%)	44 (1%)

^1^Including blank types of retouched artifacts.

Rounded percentages are given in brackets.

#### Reduction of cores

**Fig 6:**
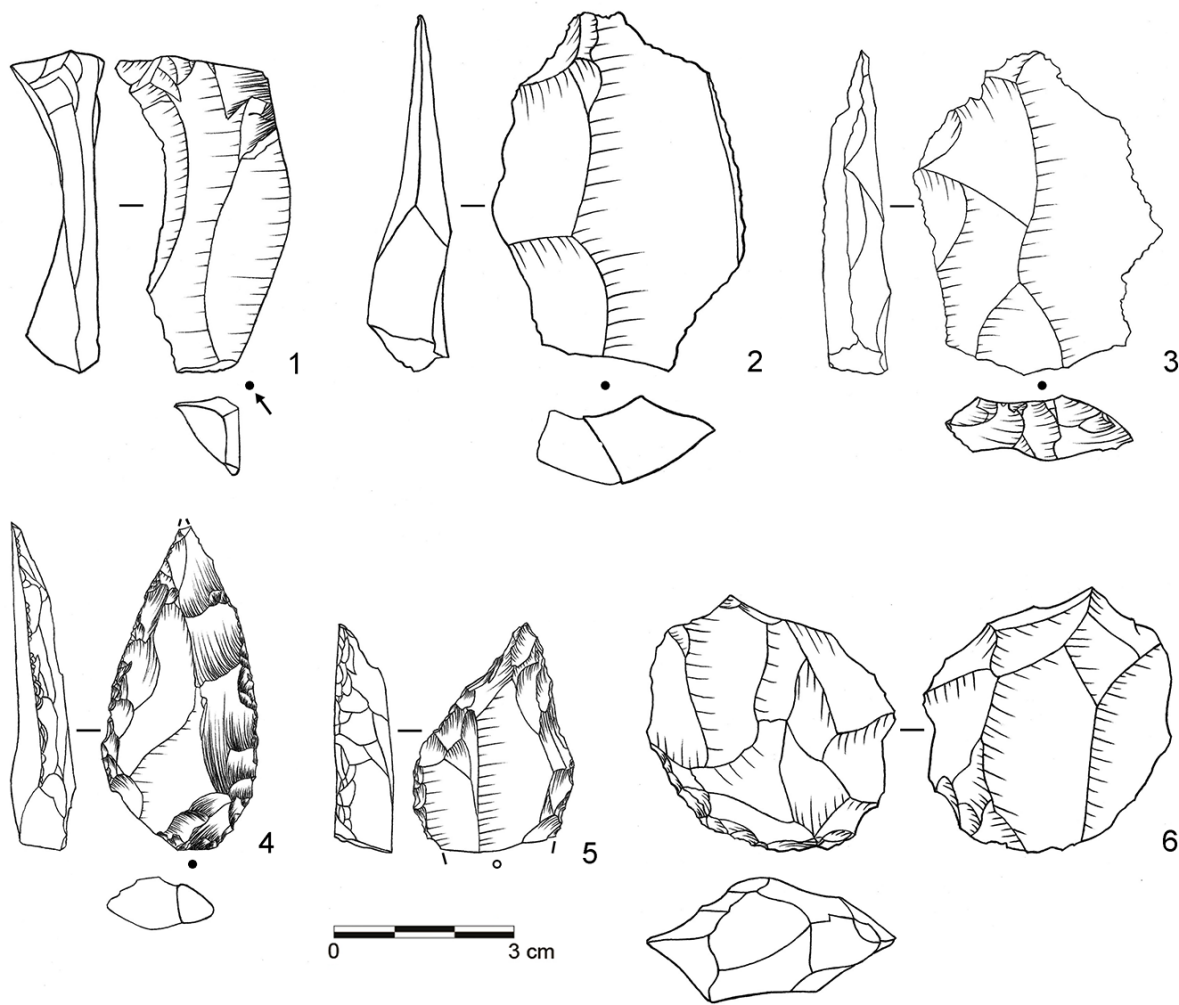
Selection of stone artifacts from assemblage BP. 1: Overshot flake from unidirectional platform blade core (dolerite, D3-955); 2: Levallois flake with partial core edge (dolerite, D3-922); 3: Levallois flake (dolerite, C3-896); 4: Unifacial point, Ndwedwe (hornfels, C3-951); 5: Asymmetric unifacial point, ACT (dolerite, C3-891); 6: Inclined core (dolerite, C3-929). (Drawings by L. Brandt and M. Lajmiri)

**Fig 7:**
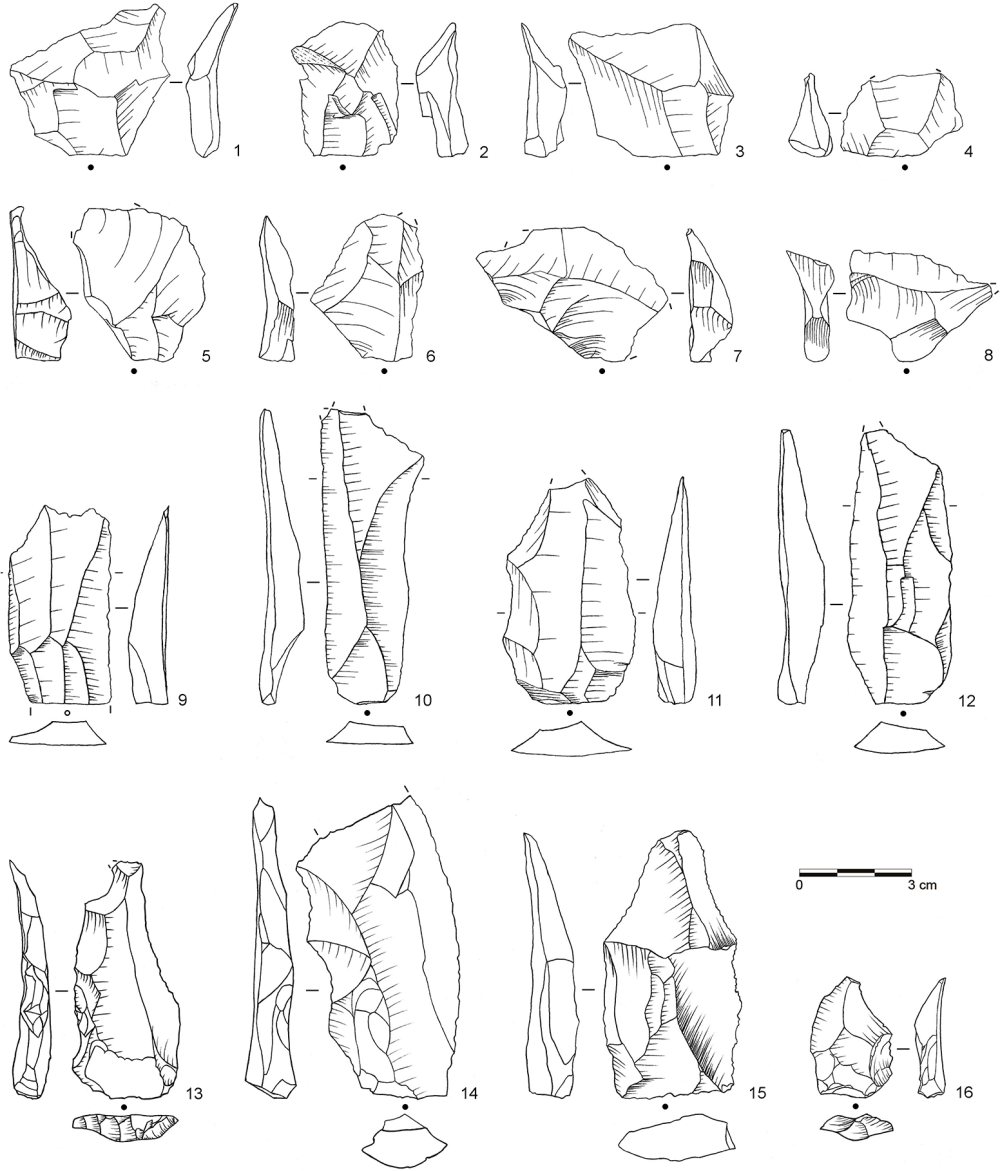
Selection of blanks from assemblage POX. 1–2: Inclined flake, invasive (both dolerite, E3-824, D3-599.4); 3–4: Inclined flake, central (both dolerite, E2-555, E2-638); 5–8: Inclined flake, partial core edge (all dolerite, E3-939, E3-973, D2-450, E3-909); 9: Blade, bidirectional (hornfels, D2-592); 10–11: Blade, bidirectional (dolerite, D3-600, E3-871); 12: Blade, orthogonal (E2-485); 13–16: Levallois flake (all dolerite, C2-934, D2-627, D2-646, D2-610.10). (Drawings by L. Brandt and M. Lajmiri)

**Fig 8:**
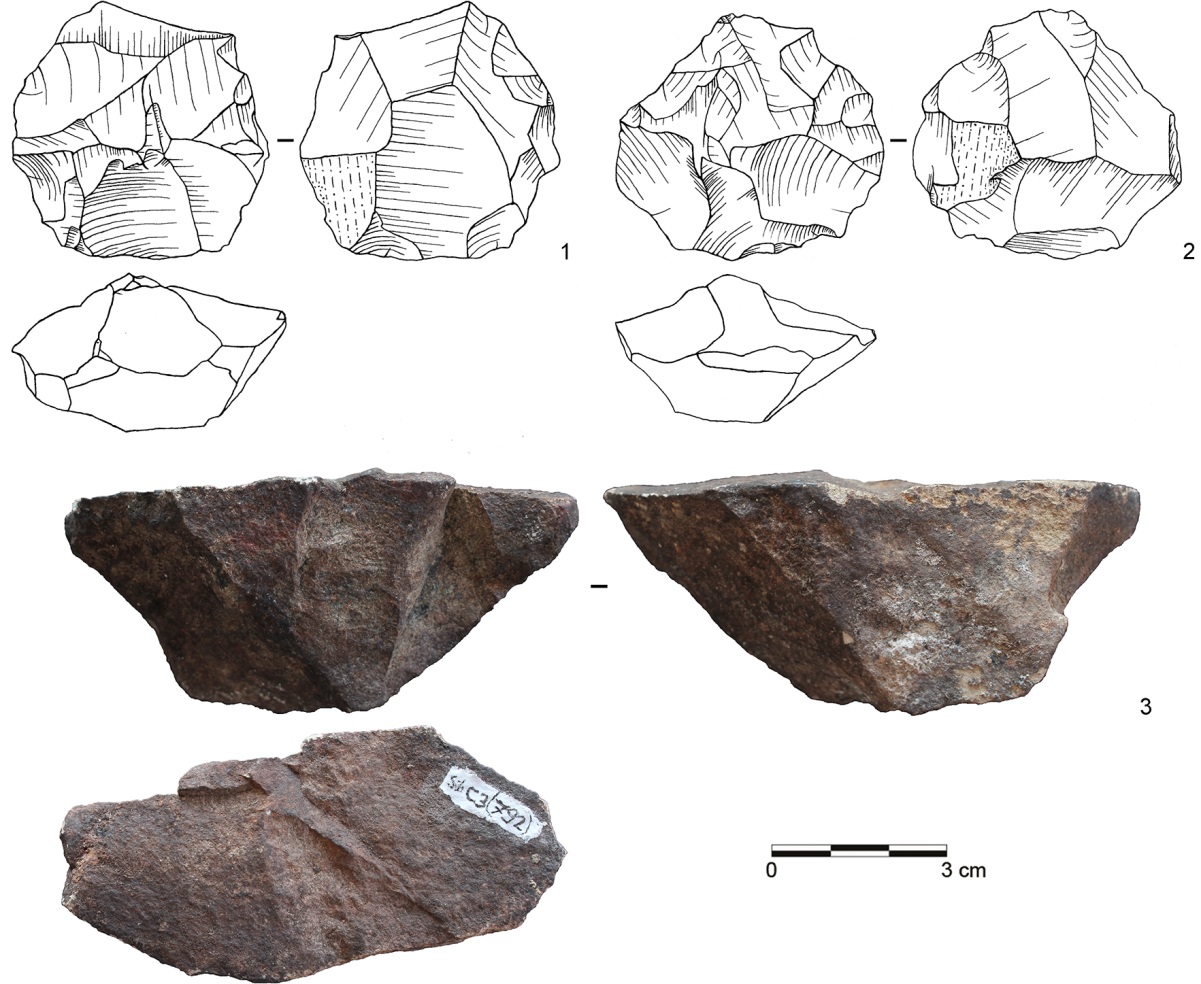
Selection of cores from assemblage POX. 1–2: Inclined core, bifacial (both dolerite, E2-700; E2-708); 3: Single-platform core, blades (dolerite, C3-792). (Drawings by L. Brandt and M. Lajmiri; photograph by J. Becher)

**Fig 9:**
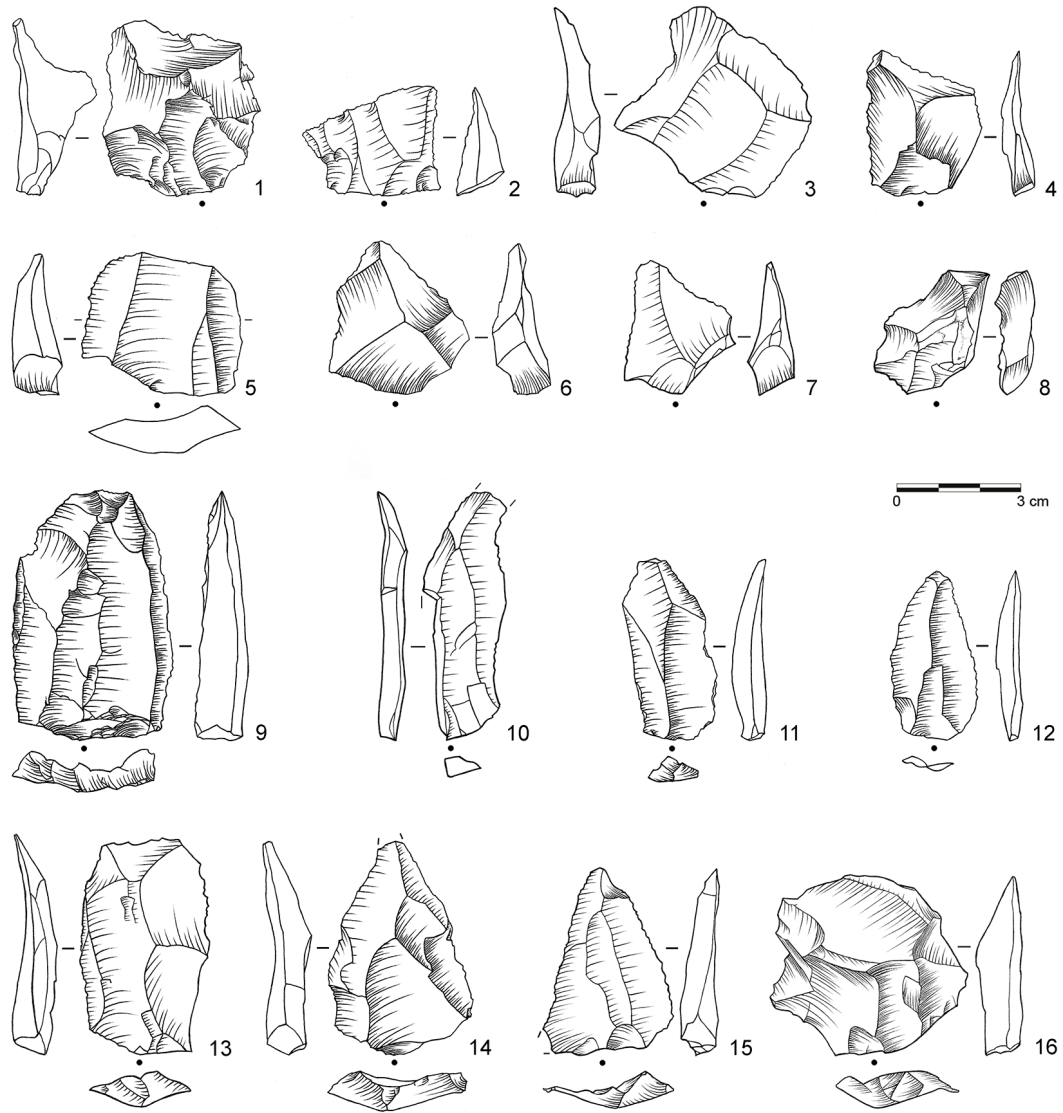
Selection of blanks from assemblage SU. 1: Inclined flake, invasive (dolerite, D3-1111); 2: Inclined flake, central (dolerite, E2-852); 3–4: Inclined flake, partial core edge (both dolerite, C3-1022, E2-947); 5–8: Inclined flake, partial core edge (all dolerite, C2-1236, D3-1222.5, D2-791, D2-838); 9: Laminar flake, bidirectional (dolerite, D3-1184); 10: Blade, unidirectional (dolerite, C2-1091); 11–12: Blade, bidirectional (both dolerite; C2-1005.3, C2-1183); 13: Levallois flake, unidirectional (dolerite, C3-982); 14–15 Levallois point (both dolerite, C2-1234, E2-934); Levallois flake, centripetal (dolerite, C2-1220). (Drawings by M. Lajmiri)

**Fig 10:**
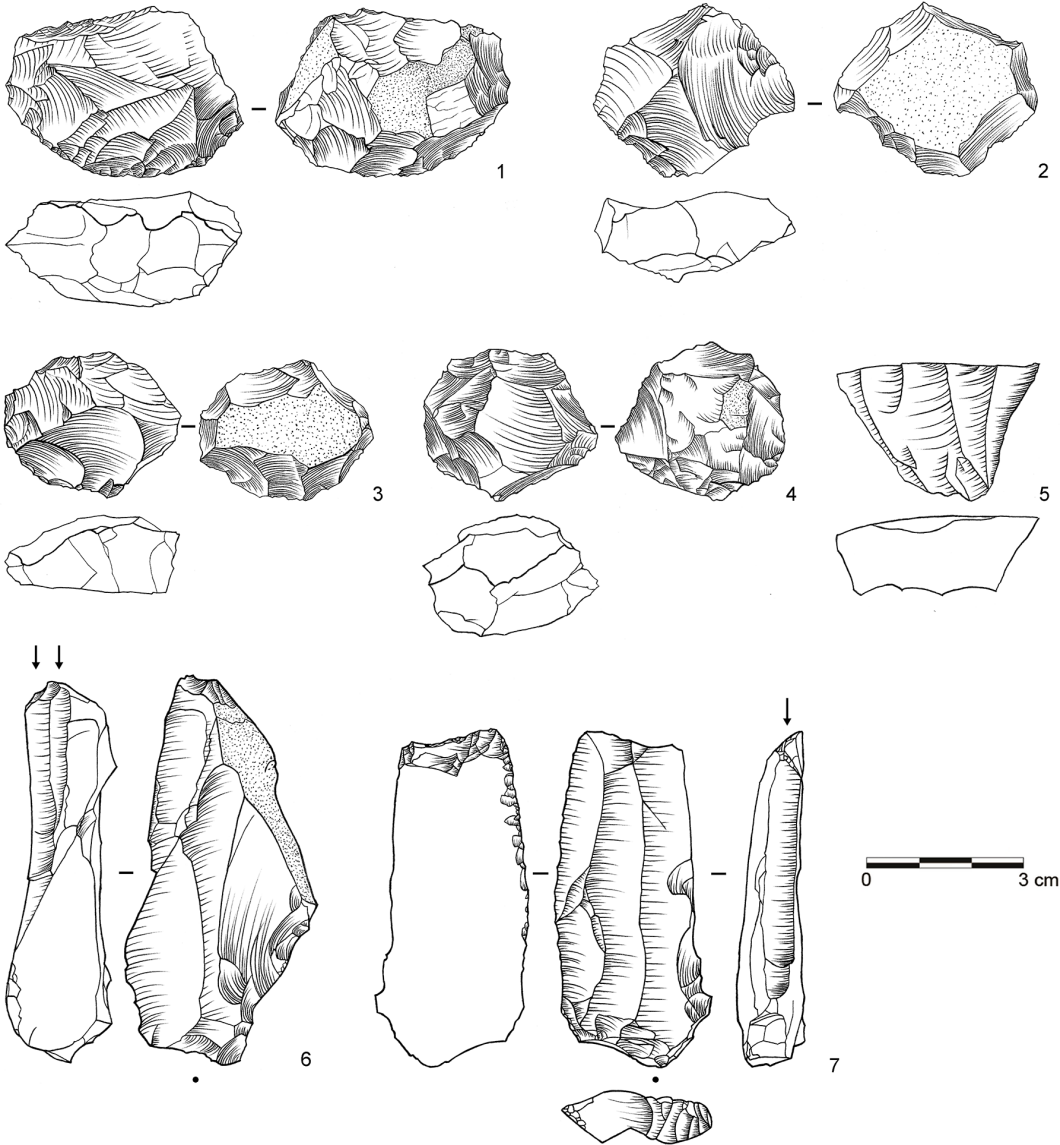
Selection of cores from assemblages SU. 1: Parallel core, bidirectional (dolerite, E2-854); 2: Parallel core, centripetal (dolerite, D3-1082); 3: Parallel core, unidirectional (dolerite, E3-1246.1); 4: Inclined core, bifacial (E3-1214); 5: Single-platform core, blades (dolerite, C2-1192); 6: Single-platform core on flake, bladelets (hornfels, C2-1127); 7: Burin on tool, bladelets (hornfels, C2-1026). (Drawings by M. Lajmiri).

**Fig 11:**
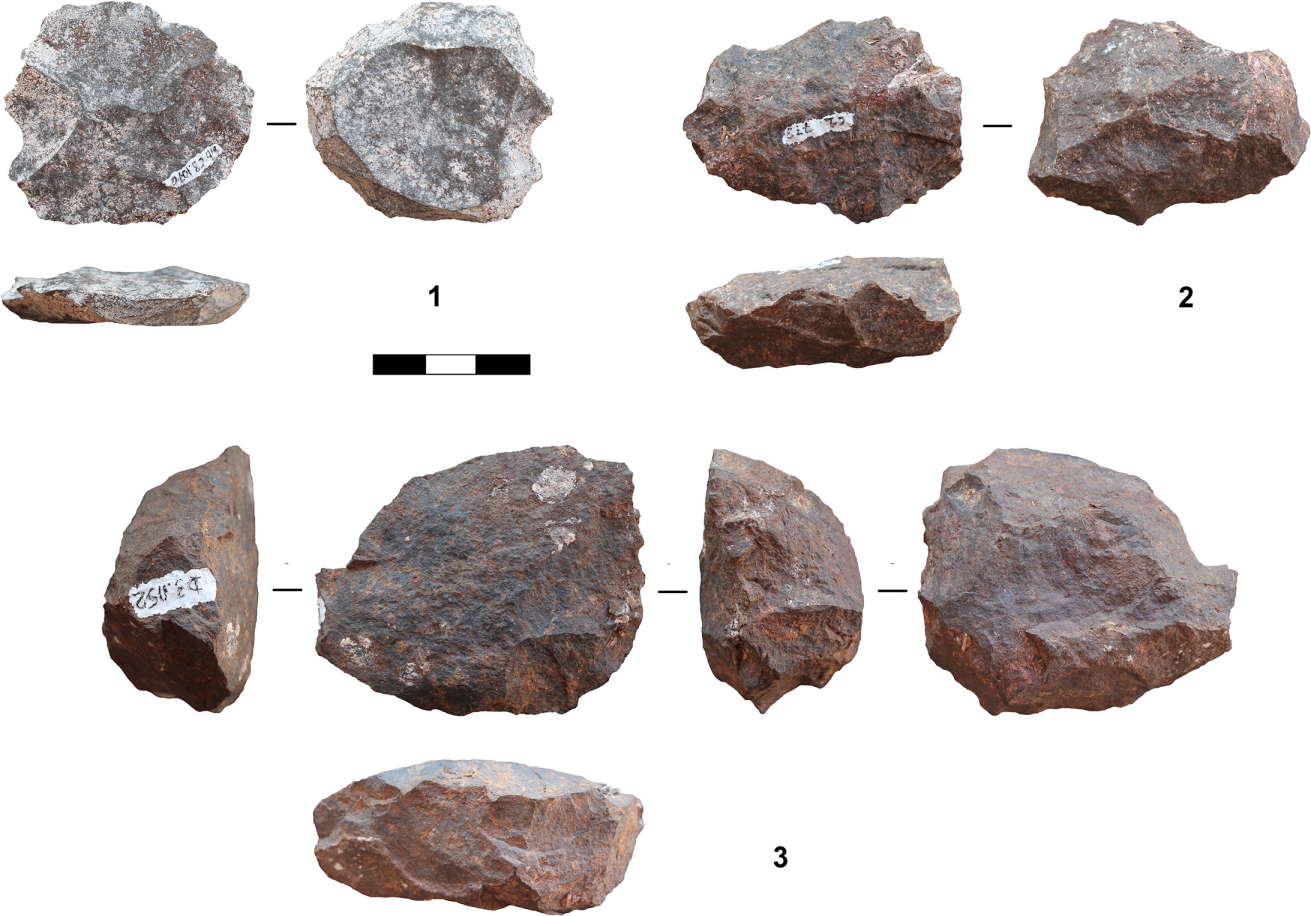
Selection of cores from assemblage SP. 1: Parallel core, centripetal (dolerite, C3-1070); 2: Parallel core, centripetal (dolerite, C2-773); 3: Single platform core, flakes (dolerite, D3-1152.3). (Photographs by J. Becher)

The methods of core reduction constitute an integral part of the technological system of MSA knappers. We chose the unified taxonomy by Conard *et al*. [71] to provide a comparable framework of core classification. We focus on the differential frequency and thus variation in use of reduction strategies, since one can commonly identify multiple knapping strategies in a single assemblage (e.g. [53] p.120). Due to the scarcity of cores in each assemblage (Table 5) and their intense degree of exploitation, the discussion of reduction strategies also draws heavily on information gained from the morphology, geometry and dorsal scar configuration of debitage products. Due to the problem of equifinality in lithic reduction, many debitage products cannot be unambiguously attributed to a specific reduction system. We thus base the following discussion on specific forms that could be directly and repeatedly associated with a particular core reduction system. While these observations cannot provide precise quantitative data, they do inform on the absence or presence of certain core reduction systems as well as their relative abundance within the studied sequence. Table 6 summarizes these observations. There are three principle methods of core reduction which vary in abundance: Parallel, platform and inclined. Figs 6–11 provide and overview for the cores and products of these systems for the newly described assemblages WOG1-POX (see [32]: Figs 10 and 11 for cores from BM-BSP).

**Table 5:** Distribution of core categories^1^.

Layer	Parallel	Platform	Inclined	Bipolar	IBR^1^
BSP	6	5	2	3	3
SPCA	8	4	-	-	-
CHE	2	1	-	-	-
MA	-	1	-	-	-
IV	5	7	1	-	1
BM	2	1	-	-	-
POX	1	4	3	-	4
BP	-	-	1	-	-
SU	5	3	1	-	2
SP	3	2	-	1	-
WOG1	1	1	-	3	-
Total	33	29	8	7	10

^1^Core classification follows the taxonomy of Conard *et al*. [71]; IBR = indeterminate broken.

**Table 6:** Relative prevalence of the main methods of core reduction within the sequence^1^.

Layer	Parallel (Levallois)	Platform (Laminar)	Inclined (Discoid)
BSP	++^2^	++	+
SPCA	++	++	-
CHE	+	+	-
MA	+	+	-
IV	++	++	-
BM	++	++	+
POX	-	++	++
BP	-	++	++
SU	+	++	++
SP	++	-	+
WOG1	++	-	+

^1^Summary based on observations from the entire lithic assemblage, including the frequency of both cores and debitage products typical for one of the reduction systems

^2^Code of prevalence: ++ = frequent; + = common;− = rare.

Parallel methods at Sibudu mostly follow a Levallois system of reduction (*sensu* [43, 88]). The cores exhibit two hierarchical and non-interchangeable surfaces and the major removals are executed parallel to the plane that intersects the two surfaces from prepared striking platforms (Fig 10: 1–3; Fig 11: 1–2). Using this method, knappers produced large rectangular, oval or convergent flakes often with facetted platforms, flat longitudinal profiles and exterior platform angles (EPA) around 90° (Fig 6: 2–3; Fig 7: 13–16; Fig 9: 13–15). In most cases, the reduction of parallel cores proceeded by unidirectional or centripetal removals with a few cores showing preferential or bidirectional modalities. Based on the number of cores and specific products, knappers used parallel production predominantly in BM-BSP and WOG1-SP (Table 6). The assemblages that lie in between (SU-POX) show a low prevalence of this method, but with some diagnostic Levallois flakes.

The second reduction system comprises various platform approaches [71, 89–91]. At Sibudu, knappers set up one or multiple platforms and removal surfaces to produce either blades or flakes (Fig 8: 3; Fig 10: 5; Fig 11: 3). The organization of multi-platform cores encompasses 2–3 platforms either positioned adjacent or opposed to one another. The platforms themselves are mostly plain, sometimes prepared and rarely cortical. Knappers mainly detached blanks from one removal surface, but cores with two or three removal surfaces also occur. The removal surfaces were located on both the broad and the narrow faces of the cores. Platform methods were particularly used for the manufacture of blades. Most of the blades from the optimal phase of debitage show plain or facetted platforms with recurrent unidirectional removals on their dorsal surfaces (Fig 6: 1; Fig 9: 10, 12), but bidirectional patterns also occur in low numbers (Fig 7: 9–12; Fig 9: 11). There are total of 7 platform bladelet cores throughout the sequence, typically with one or two plain striking platforms and one removal surface (Fig 10: 6; see also [32]: Fig 11). Blades and platform cores are most common throughout BM-BSP and are still frequent in the underlying assemblages SU-POX (Table 6). The deepest assemblages SP and WOG1 yield little evidence for this core reduction strategy.

Knappers used an inclined approach to produce flakes of various morphologies, similar to a discoid method (*sensu* [88, 92, 93]) in which removals are inclined relative to the plane defined by the intersection of the two non-hierarchical core surfaces. Two cores from layer POX exemplify this strategy at Sibudu (Fig 8: 1–2; see also Fig 6: 6; Fig 10: 4): Knappers detached flakes with an inclined angle in an alternating bifacial manner from two interchangeable surfaces of the core. This strategy lead to conically shaped surfaces and involved no preparation of platforms, as suitable angles are created by the previous inclined removals. Unifacial inclined cores with only one removal surface occur rarely. Knappers removed flakes either along the lateral edges of the core or in centripetal pattern. The diagnostic products from the edges include core edge flakes (or *éclat débordant*; Fig 7: 5, 8; Fig 9: 8) and partial core edge flakes (or *dos limité*; Fig 7: 6–7; Fig 9: 3–7). Thick central flakes with triangular or quadrangular shape and EPAs <80° (Fig 7: 3–4; Fig 9: 2) and invasive flakes with centripetal negatives removing the conical surface of the core (Fig 7: 1–2; Fig 9: 1), derive from the central centripetal reduction. The blanks detached by this strategy are smaller and lighter, but often thicker and with lower EPAs, than those of the parallel production. Specific products and cores of the inclined strategy are most frequent in the middle of the studied sequence (POX-SU; Table 6). Knappers employed discoid reduction less often in the layers below (WOG1-SP) and only rarely or not at all in the upper part of the sequence (BM-BSP).

Throughout the sequence we found only circumstantial and discontinuous evidence for cores on flakes, the use of burins for the production of bladelets (Fig 10: 6–7; n = 5; in SPCA, BM, POX and SU) and bipolar technology. Bipolar cores occur at the top (BSP; n = 3) and bottom of the sequence (SP and WOG1; n = 4), most often on quartz (5/7). It is also the quartz blanks that show most frequent traces of bipolar percussion.

#### Knapping technique

The technical act of detaching a flake from a core constitutes another major variable in technological behavior [94]. We previously described a dichotomy in knapping techniques in layers BM-BSP [32]. Knappers often produced convergent flakes with internal percussion using hard stone hammers, whereas they tended to detach blades with a soft stone hammer. We followed the same analytical procedure to examine assemblages WOG1-POX (see [32] Tab. 6). Our results suggest that the inhabitants followed similar approaches throughout the entire sequence. Flakes and convergent flakes in WOG1-POX exhibit frequent and well-developed bulbs, few proximal lips and shattered bulbs, thick platforms (average = 6.0 mm, mode = 4.0 mm; n = 2927), abundant longitudinal breaks and EPAs clustering around a modal value of 90°. These observations are consistent with percussion by a hard stone hammer a couple of millimeters away from the core edge (internal percussion; *sensu* [94]). Blades, on the other hand, exhibit fewer and generally less-developed bulbs, moderate occurrence of lips (10–25%), abundant shattered bulbs (>40%), EPAs with modal values close to 85° and thick platforms (average = 4.6 mm, mode = 3.0 mm, n = 394). We also found frequent contact points of the hammer on the striking platform, identifiable by a semi-circular break of the internal delineation of the platform and crushing in this circumscribed area. These observations suggest that knappers predominantly used soft stone hammers with internal percussion for blade production [94–96].

While the inhabitants employed consistent knapping techniques, they varied their approach to platform preparation. WOG1 and SP show comparably high values of platform facetting (23%) similar to the uppermost layers BM-BSP (22–29%), with many platforms having several facets. In between these assemblages, SU-POX show consistently less platform preparation (12–16%) and most platforms exhibit three or fewer facets. This difference can be related to the less frequent use of parallel core reduction methods in SU-POX, which involve a larger degree of core preparation compared to platform or inclined strategies.

#### Techno-economic measures

Diachronic comparisons of three independent measures regarding reduction intensities converge to the same picture. In terms of blank to tool ratio [55], total core mass to total assemblage mass ratio [56] and average thickness and length of blanks and cores [54, 55], SU-POX produce the most reduced signature (S2 Table). The uppermost layers BM-BSP exhibit far lower reduction intensities, with MA being an outlier due to low sample size. The deepest assemblages WOG1 and SP lie in between these broad patterns. Studying the flaking efficiency of an assemblage can corroborate analyses of reduction intensities as it measures the efficiency by which knapping strategies convert a mass of stone into flake edge [58, 97]. To eliminate the potential impact of different raw materials on this measure, we analyzed flaking efficiency for dolerite only in each assemblage. The results mirror the findings from the reduction intensities. Knappers used dolerite during SU-POX in the most efficient manner, followed by WOG1-SP. Above POX, there is a consistent decrease in flaking efficiency (S3 Table).

**Fig 12:**
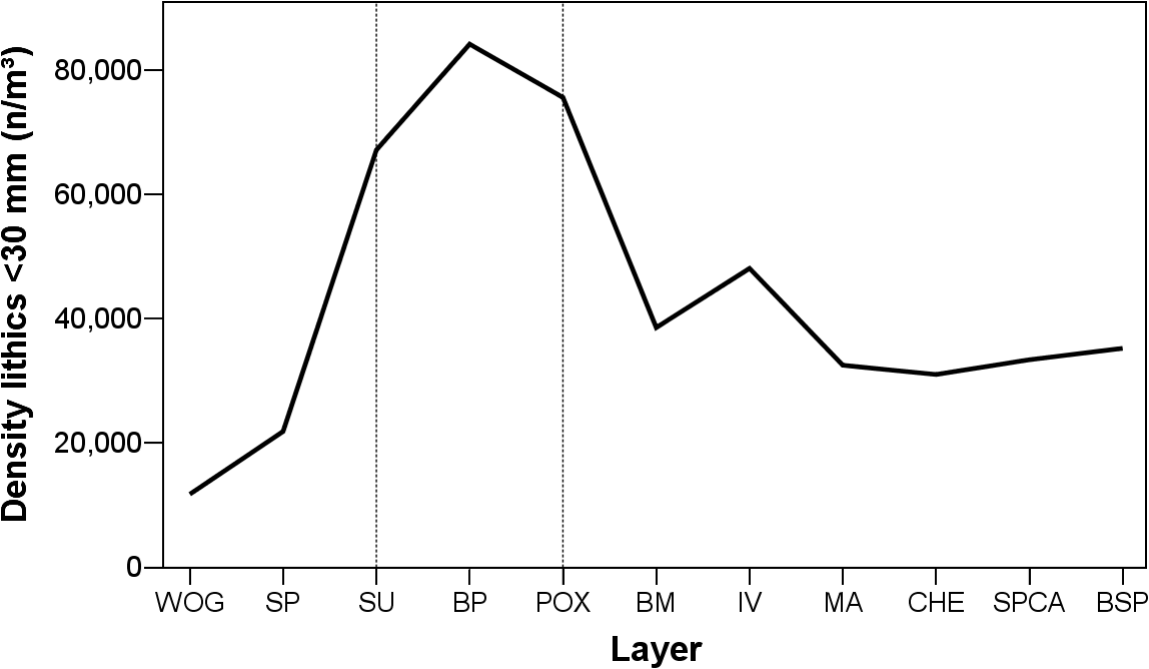
Lithic density (n/m^3^) of stone artefacts (<30 mm) throughout WOG1-BSP. Sample sizes for each assemblage can be found in Table 1. WOG1 = oldest layer; BSP = youngest layer.

Calculation of lithic find densities (n/m^3^) provides a third independent line of evidence to reconstruct techno-economic behavior, with the assumption that higher values reflect more knapping activities and artifact discard taking place on site. Total lithic densities at Sibudu range between ca. 14,000–90,000 n/m^3^, very high values compared to the few other published figures for MSA occupations (see [98, 99]). There are, however, no published density values from sites with comparable environmental setting, taphonomic context or site type which complicates a comparative assessment of settlement intensity between MSA localities based on this measure alone. In terms of intra-site patterns (Fig 12), SU-POX demonstrate by far the highest densities for small lithic finds (<30 mm; 67,000–84,000 n/m^3^). The upper six layers BM-BSP (31,000–48,000 n/m^3^) come in second, followed by the bottom strata WOG1-SP (12,000–22,000 n/m^3^). Combining all independent observations, the inhabitants of Sibudu reduced their tool stones most intensely and most efficiently during the formation of the layers SU-POX. This techno-economic behavior resulted in the highest density of lithic finds from these layers within the occupation sequence.

### Tool assemblages: Typological, technological and techno-functional analysis

**Fig 13:**
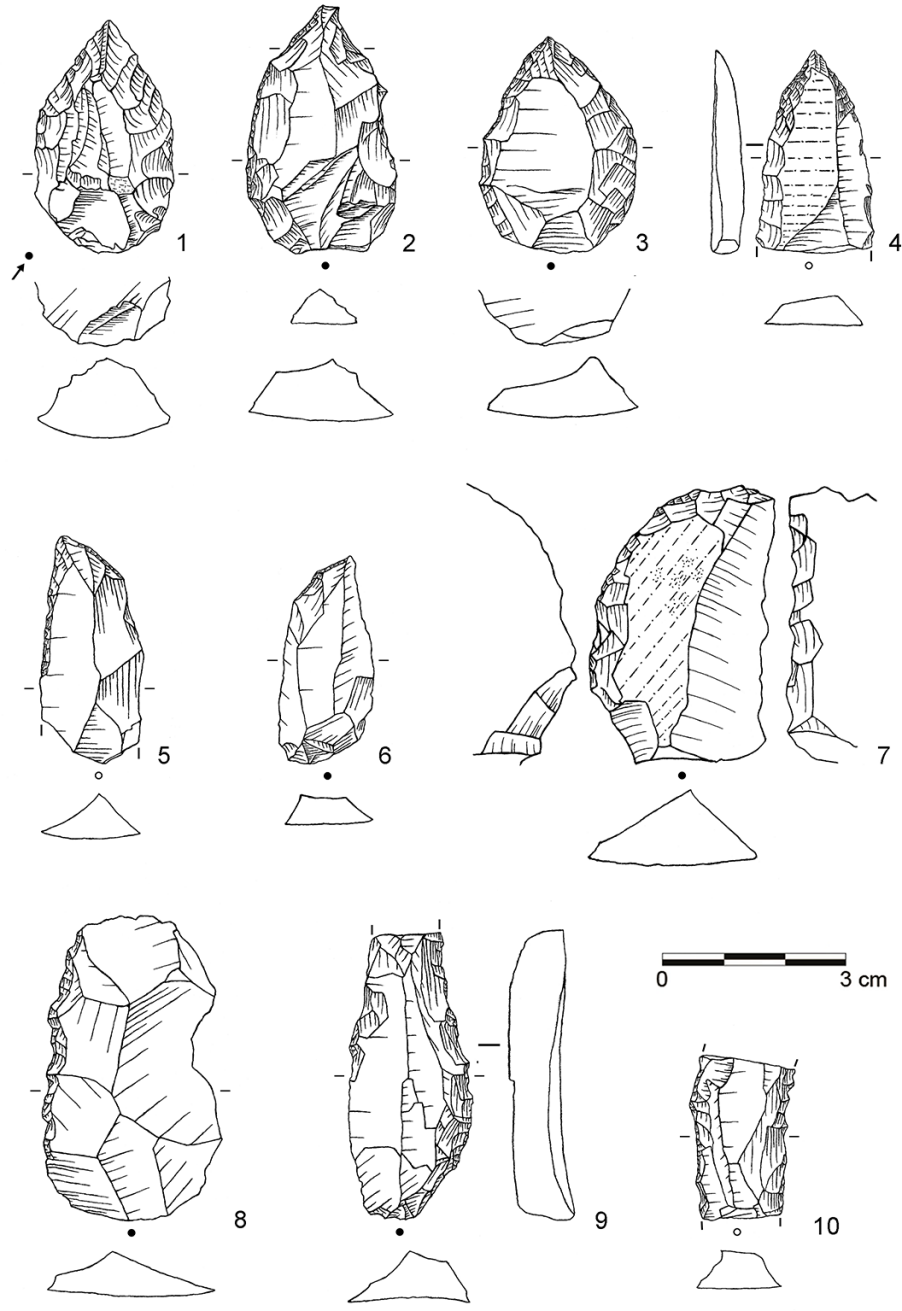
Selection of tools from assemblage POX. 1–3: Unifacial point, Tongati (all dolerite, C3-868, D2-596, C2-763.7); 4: Unifacial point, Tongati (hornfels, E3-977); 5: Unifacial point, ACT (hornfels, C2-717); 6–7: Unifacial point, ACT (both dolerite, D3-856; D3-615); 8) Denticulate (dolerite, D2-446); 9: Lateral retouch, Ndwedwe (dolerite, D3-619); 10: Lateral retouch, Ndwedwe (hornfels, D3-608). (Drawings by L. Brandt)

**Fig 14:**
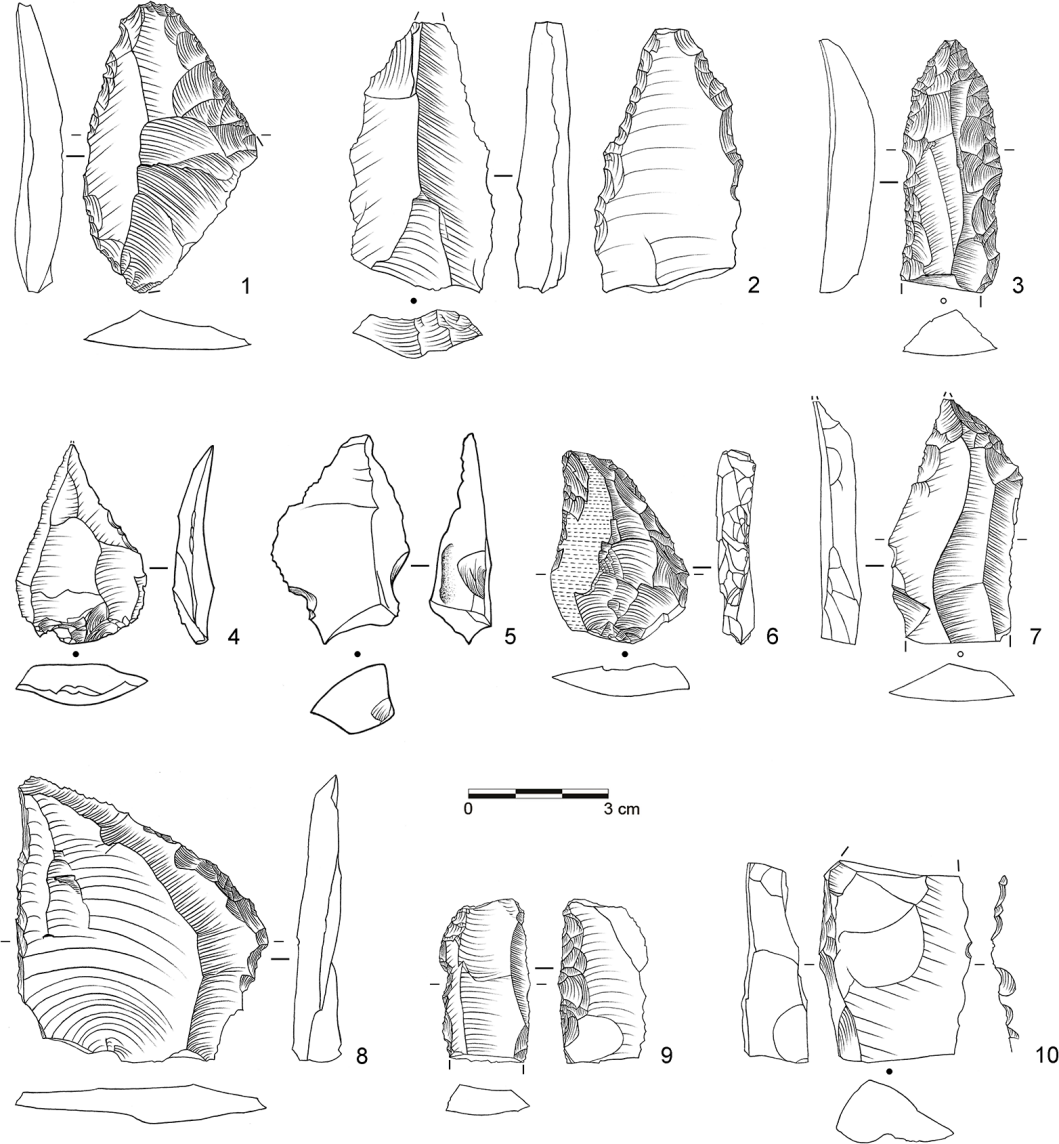
Selection of tools from assemblage SU. 1–2: Unifacial point, Tongati (both dolerite, C3-999, C3-991); 3: Unifacial point, Ndwedwe (dolerite, D3-1036); 4: Unifacial point (dolerite, E3-1092); 5: Unifacial point, notched (dolerite, E2-922); 6–7: Unifacial point, ACT (both dolerite, C3-1032, C2-1012.18); 8: Transverse scraper, NBT (dolerite, C3-996); 9: Lateral retouch (dolerite, D3-1078); 10: Denticulate, NBT (dolerite, C3-979.1). (Drawings by L. Brandt and M. Lajmiri)

Retouched artifacts from the newly described assemblages BP, POX and SU are illustrated in Figs 6, 13 and 14 (for BSP-BM, see [32]: Fig 3–5 and 13). From a traditional typological point of view [64, 65], and taking southern African MSA taxonomy into consideration [85, 86, 100], several varieties of unifacial points constitute the most abundant tool type in the studied sequence (n = 347; 45%; Fig 6: 4–5; Fig 13: 1–6; Fig 14: 1–7). They are followed by side scrapers (n = 118; 16%; Fig 14: 8), lateral retouch on blades (n = 62; 8%; Fig 14: 9–10) and denticulates and notches (n = 55, 6%; Table 7, Fig 13: 9–10). Backed pieces and bifacial points occur rarely. There are consistent diachronic changes within WOG1-BSP in overall retouch frequencies (see above) and tool composition (Table 7). In the bottom layers WOG1 and SP, unifacial points and side scrapers occur only in low numbers or not at all, whereas denticulates and notches constitute the most abundant tool type (40–78%). In the following assemblages SU and POX, unifacial points (27–37%) and side scrapers (7–23%) become more frequent and notched retouch decreases (13–19%). The youngest assemblages BM-BSP are characterized by very high proportions of unifacial points (38–54%), many side scrapers (7–23%) and few if any denticulates and notches (0–6%).

**Table 7:** Distribution of traditional tool types.

Layer	Unifacial Point	Side Scraper	Lateral Retouch	Denticulate & Notch	End Scraper	Backed tool	Bifacial Point	Hammer-stone	Minimal retouch	Other
BSP	67 (47%)	24 (18%)	14 (10%)	4 (3%)	2 (1%)	2 (1%)	2 (1%)	2 (1%)	19 (13%)	7 (5%)
SPCA	48 (46%)	24 (23%)	6 (6%)	4 (4%)	3 (3%)	-	-	2 (1%)	9 (9%)	8 (8%)
CHE	11 (38%)	7 (24%)	3 (10%)	-	-	-	-	-	8 (28%)	-
MA	26 (54%)	10 (21%)	3 (6%)	-	1 (2%)	-	-	-	5 (11%)	3 (6%)
IV	97 (54%)	23 (13%)	15 (8%)	11 (6%)	1 (1%)	2 (1%)	1 (1%)	1 (1%)	20 (11%)	8 (4%)
BM	29 (52%)	7 (12%)	6 (11%)	-	1 (2%)	-	-	-	10 (18%)	3 (5%)
POX	49 (37%)	9 (7%)	9 (7%)	17 (13%)	1 (1%)	2 (1%)	-	-	42 (31%)	4 (3%)
BP	5 (62%)	1 (13%)	-	-	-	-	-	-	2 (25%)	-
SU	14 (27%)	12 (23%)	4 (8%)	10 (19%)	-	-	-	-	11 (21%)	1 (2%)
SP	-	-	2 (22%)	7 (78%)	-	-	-	-	-	-
WOG1	1 (20%)	1 (20%)	-	2 (40%)	-	-	-	-	1 (20%)	-
Total	347 (45%)	118 (16%)	62 (8%)	55 (6%)	9 (1%)	6 (1%)	3 (0%)	5 (1%)	127 (17%)	34 (5%)

Rounded percentages are given in brackets.

Notwithstanding the low sample sizes of tools in some layers (WOG1, SP and BP), knappers reorganized their approach towards transforming blanks throughout the sequence. Whereas single-layered notched and marginal retouch prevails in the lower assemblages, multi-layered and invasive retouch that frequently reshaped the original morphology of the blanks characterizes the upper layers. Together with the overall proportion of tools and retouch debitage, as well as the diminished diversity in tool forms, these data suggest that inhabitants at Sibudu put less effort and time into on-site tool production and recycling during the lower part of the studied sequence. Regarding their different compositions, it is likely that the tool kits served different functional needs. This hypothesis will be further investigated by diachronic residue and micro-wear studies currently underway by V. Rots.

In order to examine the inhabitant’s preference for retouching certain blank types, we devised a “Blank Retouch Index” (BRI) which is computed analogous to the “Raw material Retouch Index” by Orton [101]. The index is calculated by dividing the proportion of a raw material (or blank type) among all tools by the overall proportion of the same raw material (or blank type) for all assemblages. Higher values indicate a stronger retouch preference for a particular raw material (or blank type). According to the BRI, knappers preferentially selected convergent flakes and blades to manufacture tools (S4 Table). Flakes and bladelets were less likely to be retouched. The inhabitants applied retouch almost exclusively to the dorsal side of blanks (91%) and rarely to the ventral (4%) or both (5%) faces.

The main techno-functional tool classes and reduction chains at Sibudu comprise Tongatis, Ndwedwes, naturally backed tools (NBTs), and asymmetric convergent tools (ACTs). We found an initially slow and gradual appearance of the four main tool classes throughout the sequence, followed by a more rapid increase (Table 8; S2 Fig). The oldest assemblages WOG1-SP exhibit only NBTs, whereas SU-POX features all four tool classes with combined frequencies of 33–55% (Figs 6, 13 and 14). Layer SU, the first assemblage featuring Tongatis, Ndwedwes and ACTSs, provides only low frequencies of these tools (4–6%). The four main tool classes continue to appear in the upper layers BM-BSP, but with much higher combined frequencies (67–77%), particularly for Tongatis and Ndwedwes (50–67%; see [32]: Figs 3–5). These results again demonstrate that the lowest layers WOG1-SP yield a very different signature regarding the composition of tool kits. The absence of three main techno-functional tool classes, and thus the lack of the deliberate construction of certain edge modifications of the later assemblages indicates differences in functional needs and potentially site use.

**Table 8:** Distribution of techno-functional tool classes.

Layer	Tongati	Ndwedwe	NBT	ACT	Splintered piece	Formal tool	Broken tool	Other
BSP	37 (26%)	34 (24%)	18 (13%)	9 (6%)	4 (3%)	20 (14%)	15 (11%)	5 (3%)
SPCA	30 (29%)	22 (21%)	10 (10%)	7 (7%)	4 (4%)	13 (12%)	16 (15%)	2 (2%)
CHE	9 (31%)	6 (21%)	4 (14%)	1 (3%)	-	2 (7%)	7 (24%)	-
MA	20 (42%)	12 (25%)	3 (6%)	2 (4%)	1 (2%)	4 (8%)	6 (13%)	-
IV	74 (42%)	29 (16%)	19 (11%)	14 (8%)	3 (2%)	13 (7%)	22 (12%)	4 (2%)
BM	18 (32%)	10 (18%)	6 (10%)	5 (9%)	1 (2%)	2 (3%)	13 (23%)	2 (3%)
POX	29 (22%)	8 (6%)	10 (7%)	11 (8%)	1 (1%)	30 (23%)	43 (32%)	1 (1%)
BP	3 (34%)	1 (11%)	-	1 (11%)	-	1 (11%)	2 (22%)	1 (11%)
SU	3 (6%)	2 (4%)	10 (20%)	2 (4%)	-	20 (39%)	14 (27%)	-
SP	-	-	5 (56%)	-	-	3 (33%)	1 (11%)	-
WOG1	-	-	1 (20%)	-	-	1 (20%)	3 (60%)	-
Total	223 (29%)	124 (16%)	86 (11%)	52 (7%)	14 (2%)	110 (14%)	142 (19%)	15 (2%)

Rounded percentages are given in brackets.

### Reduction sequences and use of raw materials

In previous work we observed a consistent pattern of differential raw material use between the two main tool stones of layers BSP-BM at Sibudu [32]. While both raw materials exhibit complete reduction sequences, the non-local, high-quality hornfels shows a stronger emphasis on retouch and curation, with knappers investing more energy and time in its treatment compared to the local, coarser-grained dolerite. For the layers below (WOG1-POX), we found similar trends but also differences. The strong decrease in the use of hornfels in layers SP-POX (1–6%) compared to assemblages BM-BSP, results in difficulties documenting the entire reduction sequence, in part due to the small size of these samples. Although the number of hornfels pieces is low in SU-POX, still 21–29% of this raw material are retouched, a very high percentage compared to dolerite (2–5%). Cores made from hornfels are missing (SP and BP) or very rare (n = 1; SU and POX) and hornfels blanks are predominantly non-cortical. These data suggest that the early knapping stages of hornfels took place elsewhere for assemblages SP-POX, with knappers mainly importing finished blanks and tools to the site. Dolerite, on the other hand, exhibits complete reduction sequences throughout the entire sequence WOG1-BSP. Consistent with this qualitative observation, all cortex values (0–100%) occur for dolerite in each assemblage in a gradually declining fashion (S5 Table). In contrast to the rest of the sequence, knappers reduced sandstone completely on-site in the oldest layers WOG1 and SP, including initialization, reduction of cores and tool manufacture. Quartz and quartzite exhibit mostly incomplete reduction chains, with the initial stages of knapping far better represented than the distal phases. Quartz in particular shows a high ratio of cores to blanks and tools.

We sampled a total of 15,605 small lithic products (<30 mm) from six layers by raw material, dividing the sample into 5–10 mm and 10–30 mm size classes (S6 Table). These data provide a proxy for the intensity of on-site knapping for a particular raw material. The results for both size classes mirror the observations for raw material proportions of artifacts >30 mm. There is ample evidence for on-site knapping of hornfels in the upper part of the sequence, but not in layers WOG1-BP. In contrast, knappers reduced dolerite in all assemblages, though to varying degrees, with sandstone showing a peak in the two oldest layers.

**Fig 15:**
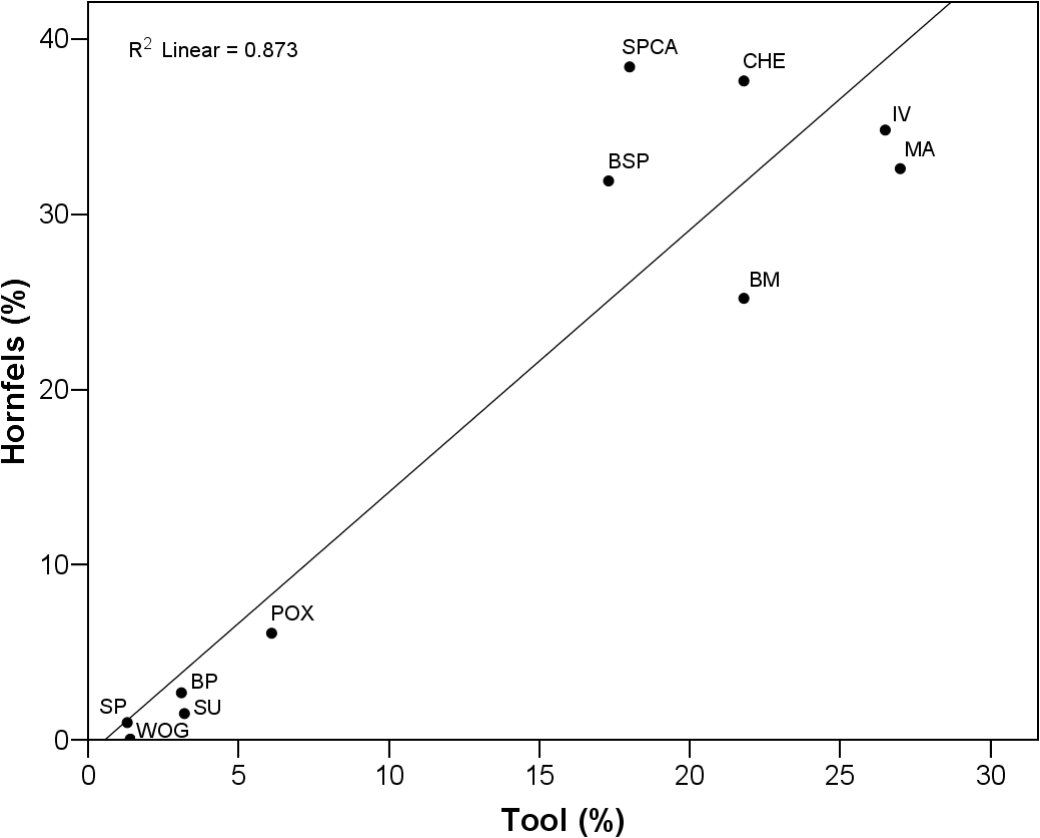
Linear regression of tool percentage and hornfels percentage of assemblages WOG1-BSP (R^2^ = 0.873; p<0.01).

The Raw Material Retouch Index shows that knappers strongly favored hornfels for the production of tools when it was in use, with blanks of this raw material being four times more likely to be retouched than those of dolerite (Table 9). The inhabitants rarely modified quartzite and sandstone. We also found a very strong and highly significant correlation between the proportion of hornfels and retouched pieces in the studied sequence (*r* = 0.934; *p*<0.001). Fig 15 demonstrates a clear separation between the upper assemblages with intense hornfels use and very frequent manufacture of tools (BM-BSP) and the lower layers characterized by low degrees of retouch and little procurement of hornfels (WOG1-POX).

**Table 9:** Raw material retouch index (RMRI) for the combined assemblages WOG1-BSP.

Raw material	Tools (n)	Tools (%)^1^	RMU (%)^2^	RMRI^3^
Dolerite	440	57.4	78.5	0.73
Hornfels	305	39.7	13.6	2.91
Quartzite	7	0.9	2.0	0.46
Sandstone	10	1.3	5.3	0.25

^1^Proportion of tools made on this raw material in all assemblages combined.

^2^Overall proportion of raw material in all assemblages combined.

^3^RMRI = Raw Material Retouch Index: Tools (%) / RMU (%) (after [101])

## Discussion

### Short-term cultural change in the high-resolution MIS 3 sequence of Sibudu

We analyzed eleven successive lithic assemblages (WOG1-BSP) from early MIS 3 deposits at Sibudu. This sequence demonstrates considerable diachronic variation in technological behavior within a high-resolution stratigraphy. The studied assemblages all date to ~58 ka [21, 22], suggesting that the behavioral changes likely took place over a period of centuries. Our study thus documents an exceptional case of short-term cultural variability during the MSA. The findings reported here stand in contrast to our results from the six uppermost layers of these deposits (BM-BSP), which yielded a homogeneous cultural signature [31, 32]. Based on the results of this study, however, we can now distinguish between three phases (S7 Table). From bottom to top these are:

- WOG1 and SP: The lithic technology is characterized by the procurement of local raw materials with the most frequent use of sandstone and an absence of non-local tool stones. Knappers invested little effort and time in making tools, but they produced predetermined convergent flakes with the dominant use of parallel methods. Platform preparation and the manufacture of blades played only a minor role. The tool assemblages exhibit little diversity in implements and consist mostly of notches and denticulates, with a near-absence of unifacial points. Only NBTs occur as techno-functional tool class characteristic of the Sibudan. Lithic find densities are comparably low and knappers did not reduce their raw materials in a particularly intense manner.
- SU-POX: The occupants of Sibudu predominantly collected and reduced the local dolerite in these assemblages, with low but gradually increasing proportions of non-local hornfels. Core reduction proceeds most often by an inclined method with little core preparation but high output of small blanks. Blades become an important blank type, produced by various platform methods. Tool frequencies are low but increase gradually, with more frequent unifacial points and a decrease in notched and denticulated implements. Tongatis, Ndwedwes and ACTs appear for the first time. They begin in low frequencies and increase considerably through this part of the sequence. Knappers reduced their raw materials in the most intense way, associated with inclined reduction strategies which produce a larger number of small flakes per core, a behavior that also resulted in the highest lithic densities within the sequence.
- BM-BSP: The assemblages exhibit a strong emphasis on the use of non-local hornfels combined with continued exploitation of dolerite. The inhabitants of Sibudu invested more time and energy in the production and curation of tools, with high retouch proportions and a diverse tool kit, as well as in the preparation of cores. Platform methods for the production of blades dominate, followed by the use of parallel systems, with little reduction by inclined strategies. Various forms of unifacial points constitute the main typological signature, whereas notches and denticulates are rare or absent. The four main techno-functional tool classes occur frequently and in all assemblages. Knappers used their raw materials in a less efficient manner and produced the largest products within the studied sequence (see also [32]).

The phases represent different strategies of lithic technology, which build upon each other in a gradual, cumulative manner. The gradual trajectory of this change encompasses a continuous increase in non-local hornfels, a stronger emphasis on the manufacture of tools, as well as a rise in the abundance of unifacial points and the four main tool classes of the Sibudan throughout the entire sequence (S7 Table). Sometimes change follows a U-shaped trajectory, as is the case for lithic density, the abundance of facetted of platforms, and the abundance of dolerite in the assemblages. Having said this, variation in these aspects is never discontinuous or erratic. Shifts in important elements of lithic technology most often occur between the groups WOG1-SP/SU-POX or SU-POX/BM-BSP (see S7 Table), supporting the notion of gradual and cumulative changes over time.

While the three assemblage groups were identified based on similar frequencies as well as absence or presence of traits (S7 Table), they can also be discriminated based on statistical analyses. Chi square tests for homogeneity find significant (*p*<0.05) differences between the assemblage groups for traits pertaining to raw material selection, debitage distribution, blank production and tool manufacture (see S1 Text). Combined with observations on qualitative differences in core reduction methods and quantitative variations in lithic density, the tripartite division provides a robust diachronic framework for the technological sequence studied here.

### Examining the causes of short-term behavioral change at Sibudu

In the following, we employ an organization of technology approach [102–107], apply evolutionary theory and draw from models of cultural transmission theory [53, 108–110] to examine the causes of the observed short-term behavioral changes. We assess the limits of these explanatory models and point to alternatives that may help to clarify the picture of cultural change during the MSA sequence of Sibudu and other nearby sites dating to early MIS 3. More specifically, we investigate whether changes in environment, subsistence, demography or other socio-cultural dynamics can be invoked as causal mechanisms for behavioral change at Sibudu.

#### Organization of technology

An organization of technology approach seems a promising explanatory model as much of the variation in lithic technology at Sibudu could be related to changes in raw material procurement and mobility strategies [59–63, 81, 103, 104, 111–113], as well as site use and reduction intensities of assemblages [54–56, 97, 114, 115]. Both access to raw material and its internal properties influence the choice of knappers and the composition of lithic assemblages [55, 59, 61, 63, 116]. The frequency of the procured raw materials, and the degree of their reduction, certainly had a strong influence on the assemblage compositions at Sibudu. The extremely high correlation between the proportion of hornfels and retouched artifacts constitutes a prime example (Fig 15). Theoretical considerations from behavioral ecology [55, 59, 63, 105, 116] predict that the degree of time and energy invested in the knapping, use and curation of this material will be higher than those for local raw materials of lower quality such as dolerite or sandstone. Exactly this pattern is reflected in the higher retouch rates and smaller artifacts made from hornfels. Furthermore, and in agreement with theoretical predictions, hornfels that travelled from further away entered the site more often as finished products and tools, with an under-representation of early products of the reduction sequence.

Knappers at Sibudu had access to a constantly high supply of dolerite and other local raw materials in the direct vicinity of the site. In theory, abundance of raw material should allow hunter gatherers to discard dulled artifacts without resharpening them and simply make new ones as needed [63, 81, 116–118]. The assemblages at Sibudu should thus on average have relatively low frequencies of retouched exhausted artifacts if scheduling of movements remained constant. Interestingly, only assemblages WOG1-POX are in accordance with the theoretical models for expedient technologies. In the upper part of the sequence, and even though raw materials were constantly abundant around Sibudu, the occupants of the site increasingly chose to use non-local hornfels. This pattern stands in contrast to theoretical predictions that claim that local resources will always be used when available (cf. [119]), but does none the less help to explain high retouch frequencies in these layers with abundant hornfels. While factors such as predictability of resources and scheduling of movements in the subsistence round constitute additional behavioral dimensions driving variable use of local vs. non-local raw materials in these models [81, 104, 116–119], Sibudu so-far constitutes a single data point in the landscape, and we thus lack the necessary contextual information to evaluate these variables. We can only speculate on whether or not low predictability in access to resources and scheduling of movements in the lower part of the sequence could have led to an increased reliance on local rocks with higher reduction intensity, whereas increased predictability in the upper layers resulted in the opposite behavior. That being said, paleoenvironmental indicators, hunting behavior and principal site function remain more or less constant throughout the studied sequence (see below).

Raw material properties constitute an additional dimension that warrants consideration when comparing the techno-economic use of dolerite vs. hornfels (cf. [120]). Hornfels is easy to knap, but its sharp edges are fragile and have a higher tendency to break, rendering frequent resharpening necessary. In contrast, dolerite yields more durable edges that remain sharp for a long time, decreasing the need for retouch and resharpening [79]. We have previously hypothesized that this observation helps to explain the high percentage of tools in layers BM-BSP [32], and subsequently we have found a similar pattern at the MSA locality Holley Shelter [121]. In support of this, the abundance of tools decreases sharply in the lower occupation horizons where knappers used more durable local raw materials, such as dolerite, sandstone and quartzite.

Land-use strategies describe the pattern in which hunter gatherers move across the landscape over time, encompassing the selection of occupations, task specific locations, how they acquired resources and implemented lithic technology [103, 104, 122, 123]. The assemblages at Sibudu can be divided into those with a strong predominance of local raw materials (WOG1-POX) and those with a high percentage of non-local hornfels (BM-BSP). Examining this criterion, mobility increases during the younger assemblages, either by larger range for activities related to subsistence or more frequent relocation of camps. As explained above, shortage in local raw materials cannot explain the archaeological signatures at Sibudu and changes in the scheduling of movements in the subsistence round are difficult to assess at the site level. The change in raw material selection could, however, indicate that subsistence activities were generally carried out over larger distances during the later phases of the studied sequence. If, due to their intense exploitation, food resources around the site became depleted or less predictable, a strategy of embedded procurement during further-reaching subsistence rounds would likely lead to the greater use of non-local lithic resources, all else being equal (e.g. [103, 104, 123, 124]). While the faunal assemblages of the studied sequence show that the inhabitants generally hunted the same type and frequency of animals, there a slight increase in large-sized bovids in the topmost assemblages [125, 126]. The intentional choice of hornfels from further away via direct or special purpose procurement [124, 127] to produce specific kinds of tools, or due to other socio-cultural factors [119], may also help to explain the high frequency of non-local raw material in BM-BSP.

Based on the high density of lithic remains, the frequent occurrence of combustion features, evidence for site maintenance, numerous faunal remains, the use of ochre and the construction of bedding, Sibudu functioned as a base camp throughout the entire period under study (cf. [103, 104]). Thus, simple predictions about land-use strategies from retouch frequency and artifact density [118, 128] do not apply to Sibudu where these variables fluctuate throughout the sequence. The changing composition of tool kits–notched implements vs. unifacial points–certainly played a role in subsistence tasks, such as a higher reliance on plant or wood processing for occupations rich in denticulates [129]. While the general function of the site remained the same, these variations likely reflect task-specific changes in the frequency of various activities of daily life.

The intensity of stone artifact knapping at a site and the reduction intensity of raw materials constitute further important factors influencing the size, composition and morphology of lithic assemblages [54–56, 58, 76, 103]. The highest reduction intensities (S2 Table) and lithic densities (Fig 12) are associated with the most frequent use of dolerite (Fig 3) and inclined core methods (Table 6) in the middle of the sequence (SU-POX). The intense on-site reduction of dolerite by means of inclined reduction, with the production of large numbers of small flakes, helps to explain the highest lithic density in this part of the sequence. The more frequent use of hornfels in BM-BSP, for which part of the reduction sequence took place elsewhere, and the dominant use of prepared core strategies in WOG1-SP could explain the comparatively lower lithic densities in these groups.

From a techno-economic viewpoint, scholars have proposed that the frequent application of inclined reduction strategies, such as the discoid method, constitutes an economizing behavior as the number of usable flakes is higher in relation to prepared core strategies such as Levallois [88, 90, 130–132]. Inclined systems exploit the volume of the core through a continuous series of flakes that can be knapped without preparation, thus conserving raw material. Recent experimental studies also demonstrate that inclined concepts are less vulnerable to raw material constraints and provide a steadier output of usable cutting edge compared to some platform core technologies [131]. Values for the flaking efficiency of dolerite (S3 Table) show that knappers at Sibudu used this raw material economically during the formation of layers SU-POX. They did this presumably because other high-quality raw materials were not available in the reduced area in which they gathered and hunted. This interpretation is consistent with the fact that whereas hornfels is intensely retouched in layers BM-BSP, knappers exploited dolerite in a less efficient manner and made less use of inclined technology. These observations are in agreement with theoretical models predicting that lithic assemblages used by hunter gatherers with lower mobility will contain fewer formal tools and prepared cores [63, 116, 133–135].

In conclusion, changing patterns of mobility coupled with a different selection and use of raw materials can explain a portion of the variation in lithic technology observed at Sibudu. While Sibudu always served as base camp during the period under study, variations in retouch intensity and marked differences in the composition of tool kits document a dynamic economic context in which innovations and directed cultural change occurred.

#### Evolutionary theory and environmental forcing

Evolutionary models of cultural change constitute another candidate to interpret the data from Sibudu. In a classic evolutionary model, changes in populations of organisms are explained either by biotic or abiotic mechanisms of natural selection working on individuals and populations on various spatiotemporal scales. The interplay between abiotic and biotic ecological factors drives evolutionary change, with historical contingency determining the importance of different selective agents [136–139].

In Paleolithic archaeology and paleoanthropology, researchers often favor abiotic factors of selection to explain cultural change. Long-term changes in the environment put novel selection pressures on hominins to which they have to react by modifying their behavior and material culture in order to prosper and reproduce. Temporal correlations between environmental data and cultural change on large scales usually form the explanatory link in these models (e.g. [140–148]). Within the southern African MSA such approaches have been put forward to explain the appearance and disappearance of the SB and HP [29, 149–151] (but see [152]).

In order to apply this theoretical framework, we combined local paleoenvironmental ([37, 125, 126, 153–164]; summary in S2 Text) and subsistence data [125, 126, 161, 162] for layers WOG1-BSP at Sibudu with our own observations on lithic technology. The results show a remarkable disconnect between environmental and cultural change. Various indicators of climate and vegetation, suggesting drier and warmer conditions with more open grassland habitats than today, remain constant throughout the period of study (see [17, 126, 157–159]) with minor random fluctuations (see [160]), while the lithic technology undergoes marked and unidirectional changes. Behavioral change thus occurred independent of environmental change, suggesting that the observed alterations in technology do not constitute adaptive responses to variable natural environments. Some of the paleoenvironmental proxies, however, are not as finely resolved as the lithic data (S2 Text). While we work on achieving the same resolution for all datasets in the future, the difference in scale for some environmental proxies is a potential confounding factor of this interpretation.

Regarding subsistence, the inhabitants of Sibudu constantly hunted the same types and range of animals in each occupation horizon of the sequence WOG1-BSP, particularly large-sized bovids (ungulates). Subsistence activities, at least in terms of hunting, are thus not the main driver of different knapping strategies and the manufacture of different kinds of tools (cf. [126]). In contrast, the largest shifts in terms of environmental indicators and hunting strategies occur between layers YA2-G1 (“post-HP MSA 2”) and G1-BSP (“post-HP MSA 1” [17, 125, 126, 156, 158, 162]) and above BSP in the final MSA [159].

#### Cultural transmission theory

Due to the gradual and cumulative changes observed in the sequence at Sibudu, and the mismatch between environmental, subsistence and lithic technological data, we viewed our results from the perspective of cultural transmission theory [108–110, 165–167]. The frequencies of Tongatis, Ndwedwes, NBTs, and ACTs serve as a good example for the observed gradual and cumulative changes. As these artefacts reflect repetitive actions of knappers with the goal of producing a particular functional edge morphology by means of retouch, their reproduction by other individuals required high-fidelity social transmission of technological information. The four main tool classes start off in very low numbers (WOG1 and SU) followed by a rapid increase (BP-POX), and then a slowdown at high values (BM-BSP). This unidirectional trajectory resembles the S-shaped cumulative distribution curve that was found typical for the spread and adoption of many new technologies, practices and beliefs [108, 109, 166, 168], admittedly over much shorter timescales [169].

According to Rogers [168] and Henrich [166], such an S-shaped uptake curve usually indicates local innovations with a subsequent increase in frequency by means of biased cultural transmission via social learning instead of random drift of a neutral trait (see also [170]). Biased transmission denotes the circumstance in which populations favor certain cultural variants over others during the process of information transmission. In other words, certain cultural elements were preferably maintained and passed on to next generation due to reasons of their function, popularity, prestige, association with important individuals or ease of imitation [108, 169, 171]. In contrast to such a directed positive selection, drift of a neutral trait most often follows the pattern of an erratic stochastic process, rarely creating a unidirectional or S-shaped cumulative distribution curve [108, 109, 166, 170, 172, 173]. We also consider it highly unlikely that the specific and functionally relevant configuration of edge modifications of the techno-functional tool classes represent repeated random errors during information transmission, which would be the basis of random drift particularly in small populations (e.g. [164, 172–174]). For other traits in the studied sequence, such as non-functional or single-component elements that exhibit non-directional temporal changes in frequency, random drift remains a plausible explanation that we will examine once the entire ~58 ka lithic sequence is available.

For now, we do not know exactly why these techno-functional tool classes were transmitted to successive generations (cf. [169, 171, 175]), but we emphasize the importance of socio-economic over environmental selection factors in their appearance and distribution. Presumably these tools worked well for the purposes for which they were used and thus experienced positive selection that led to the increase in the frequency of their manufacture and use. V. Rots is currently conducting functional studies and residue analysis at Sibudu which should provide us with more specific explanatory hypotheses in the near future.

While the sequence at Sibudu shows directed behavioral change, this takes place against a backdrop of relatively stable technological adaptations. These unifying characteristics include 1) the regular collection of dolerite and its complete reduction on site, 2) the use of the same variants of quartzite, 3) the consistent application of knapping techniques and 4) the use of similar reduction methods. These observations together with the nearly continuous occupation of the site during the period of study reflect successful transmission of information between successive generations living in the region of Sibudu.

#### Demography

Changes in demography, such as migrations of people, fluctuations in population size, population composition, or general population pressure, might play a role in the observed cultural change at Sibudu, and these variables have been popular themes in research on the MSA [25–27, 176–178]. The gradual, cumulative and often unidirectional change in the lithic technology at Sibudu is consistent with cultural transmission within a shared information system. Hence, our technological observations do not require any radical changes in human populations or migrations, and we favor a model of demographic continuity over the course of the sequence under study. This being said, it is plausible that innovations from neighboring groups may have spread to the occupants of Sibudu via social and economic interaction.

In terms of population size, the available archaeological evidence suggests that all assemblages that follow the HP at Sibudu derive from intense occupations, which may reflect high local population densities [17, 21, 38, 39]. This intensification at ~58 ka may have resulted from longer visits, more visits, or larger groups than during earlier phases of occupation, but we cannot easily distinguish between these possibilities.

#### Summary

The short-term cultural variation that we have observed in the late MSA lithic sequence at Sibudu can be interpreted using both an organization of technology approach and cultural transmission theory. Standard evolutionary models based on climatic or environmental forcing are not required to explain our observations. Similarly, while the spread of ideas between neighboring groups is possible, such a diffusion of ideas is not needed to generate the changes during this phase of occupation at Sibudu. This is of considerable interest since most explanatory models for the MSA have invoked environmental or demographical causality to explain behavioral change. Unlike most other studies, however, we have presented behavioral variation during the MSA with a very fine temporal resolution. These data bring us closer to a paleo-ethnographic time scale. On such a scale, other factors become plausible causes of technological change. Here we are tracing social interaction and human behavior across life spans and generations rather than millennia, which is the more typical temporal scale of research in the MSA (see also [2, 23, 126, 179]).

## Conclusions

What are the implications of our findings for the MSA culture-stratigraphic sequence of KwaZulu-Natal and South Africa during MIS 3? While trying to answer this question, we also evaluate the role of short-term cultural change for the definition of the Sibudan [31, 32] and discuss methodological considerations from our approach.

Lithic assemblages that succeed the HP and fall within MIS 3 comprise the so-called “post-HP” [86], “MSA 3” [180] or “MSA III” [176]. We have recently criticized these units as catch-all categories that have done more to hinder rather than to stimulate research ([31, 32]; see also [181–184]). Here, we emphasize trends emerging from recent analyses of lithic technologies in eastern South Africa during MIS 3. As more and more sites are studied with comparable methods [31, 121, 179, 185, 186], technological and typological variability, rather than stasis, appears to be the main cultural signal within KwaZulu-Natal during MIS 3.

In this article, we demonstrated cultural change at one site within a narrow time frame during early MIS 3. As an example of cultural variability on the regional scale, broadly contemporary lithic assemblages from Umhlatuzana, 90 km southwest of Sibudu, feature finely made bifacial points and small backed segments [186, 187]. These tool assemblages are more similar to the HP at Sibudu [188] than to those of WOG1-BSP that we presented here. Our reanalysis of the broadly contemporaneous MSA sequence at Holley Shelter [121], located only 40 km west of Sibudu, provides some parallels but also marked differences to Sibudu. Whereas the middle of the sequence conforms to the Sibudan as characterized in Will *et al*. [32], the upper and lower parts do not match with any of the three technological groupings that we have presented here. Particularly the upper occupation horizons at Holley Shelter, characterized by a strong dominance of splintered pieces, mark a distinctive pattern of lithic technology. Slightly later in the sequence, the final MSA assemblages at Sibudu (~38 ka) and Umhlatuzana (~35 ka) feature hollow-based points as characteristic tool form that occurs exclusively in KwaZulu-Natal during late MIS 3 [181, 185, 187]. To conclude, apart from the unifying characteristic of frequent unifacial points [179, 185], assemblages following the HP in this region encompass much more diachronic and spatial variability than has been previously recognized.

The Sibudan, a techno-complex we originally defined at Sibudu based on the six assemblages BM-BSP [31, 32], constitutes a case in point. As discussed above, the two assemblage groups SU-POX and WOG1-SP show both similarities and differences to the original definition. SU-POX conform to all principle techno-typological criteria of the Sibudan with differences being gradual and quantitative, such as a lower abundance of tools in general and more specifically Tongatis and Ndwedwes. In contrast, the oldest assemblages in our study (WOG1-SP) exhibit stronger differences, such as the absence of unifacial points and three techno-functional tool classes, a lack of blade production and the exclusive use of local raw materials. Thus, while the assemblages SU-POX can be included within the existing concept of the Sibudan techno-complex without transcending its previously defined techno-typological boundaries, the lower part of the sequence raises several new questions and challenges the concept of the Sibudan. This problem is to a large extent an issue of perspective. When looking at the entire sequence, gradual and cumulative change stands out. When focusing only on the youngest and oldest assemblage of the studied sequence, techno-typological differences are the prominent pattern.

**Fig 16:**
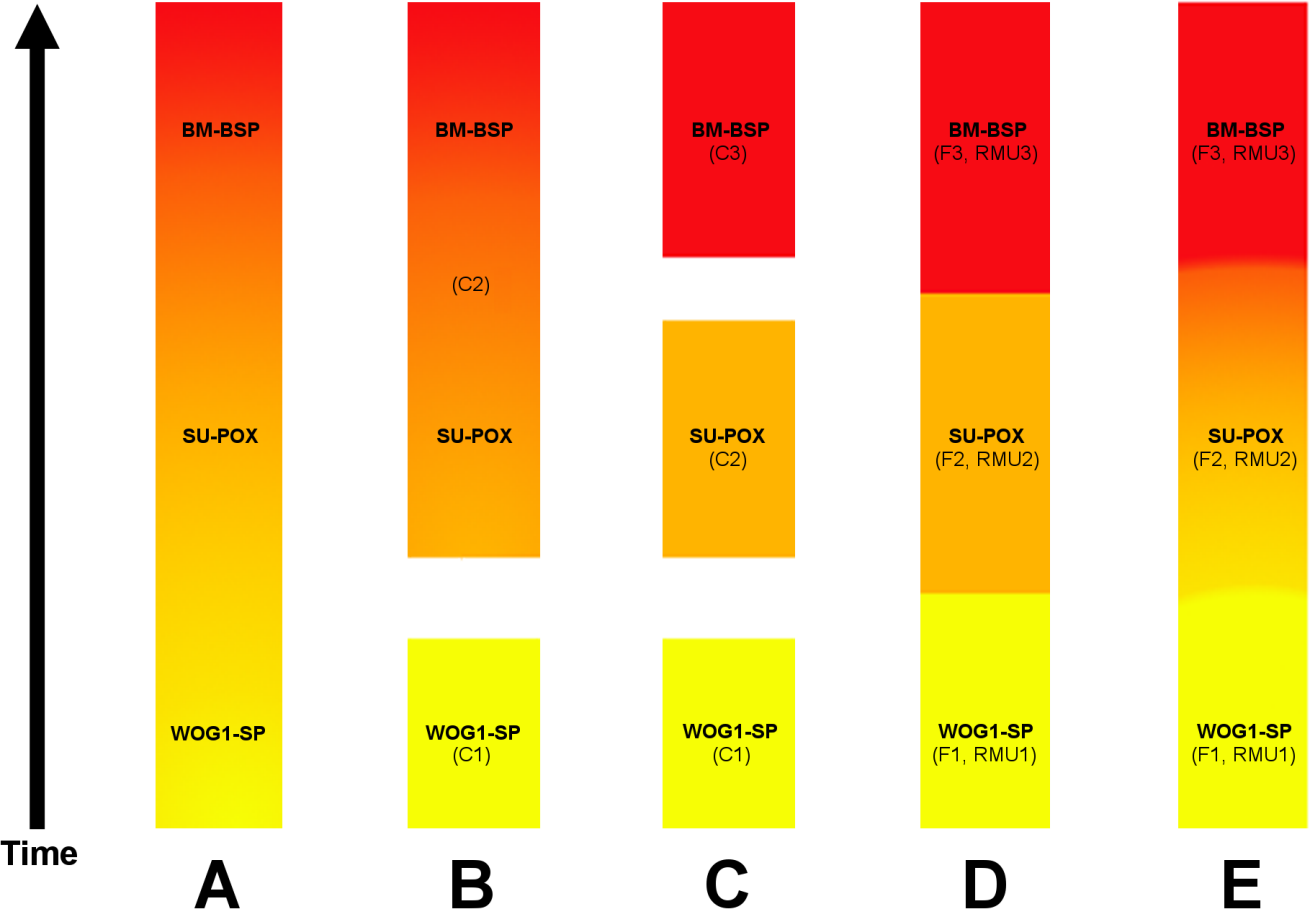
Schematic representation of hypotheses to conceptualize the short-term cultural changes at Sibudu throughout WOG1-BSP. A) Gradual change with continuous cultural transmission among local populations throughout the entire sequence (broad Sibudan definition). B) Discontinuous change with two distinct units (C_x_)–one encompassing internal gradual change–separated through disruption of information transmission or occupation hiatuses. This could either reflect two independent populations or cultural taxonomic units (SU-BSP as a narrow Sibudan definition). C) Discontinuous change with three distinct units (C_x_), separated through disruption of information transmission or occupation hiatuses. This could either reflect three independent populations or cultural taxonomic units (“splitter” taxonomy with BM-BSP as originally defined Sibudan). D) Discontinuous change with three groupings reflecting different site function (F_x_), technological organization, or raw material use (RMU_x_) at different time periods during the occupation of the locality. This hypothesis does not include statements about information transmission or population displacement. E) Gradual change with continuous cultural transmission among local populations in the region around Sibudu. Within this continuum, three groupings can be concerned based on differences in site function (F_x_), technological organization, or raw material use (RMU_x_) at different time periods during the occupation of the locality.

The central issue is how we can best view short-term cultural change over narrow time spans (Fig 16). Considering that all of these horizons are of indistinguishable OSL ages of ca. 58 ka and reflect a nearly continuous sequence of occupations, we ideally would like to place them within the same cultural taxonomic unit. Indeed, if we view time as the leading variable for defining analytical units, we must place them in the same unit. Alternatively, if we grant technology and the nature of the material culture primacy, at some point this variation goes beyond the spectrum of what we can comfortably place within the Sibudan. To complicate this situation, changes in the procurement and use of raw materials as well as site function demonstrably influenced the techno-typological characteristics of the assemblages. These and similar issues were at the heart of the various scenarios that characterized the Mousterian debate of the late decades of the 20^th^ century.

A narrow definition of the Sibudan would render comparisons with other sites easier, but may ultimately lead to complex terminology if researchers follow the approach of “splitters” to its logical conclusion. This approach would also impose arbitrary boundaries in an essentially contemporaneous sequence characterized by cumulative changes. We propose several hypotheses in Fig 16, favoring those that emphasize gradual change and continuity, but refrain from providing a final answer to this question as there are still about 0.5 m of sediments dated to ~58 ka left to study. Referring to Brew [33], there is no universally valid answer to the question of how much variability can be incorporated into one techno-complex. Answers to such questions can only be found in the context of well-defined research questions, and the utility of any cultural taxonomy can only be assessed in terms of how it helps us gain insight into past human lifeways or for testing specific hypotheses. For now, we are in the midst of a phase of more inductive research in which it is essential to establish reliable technological observations from within well controlled chronostratigraphic contexts. Future synchronic and diachronic studies on various scales will test the definition and value of the “Sibudan” as a concept for structuring the MSA record of MIS 3.

Turning back to the larger geographical scale, there are interesting technological differences between the western, southern and eastern parts of southern Africa during MIS 3. The decline in the number and intensity of occupations after the HP in the Western Cape, especially between 50–25 ka (e.g. [23, 30, 189]), finds no equivalent in the eastern part of southern Africa. Here, the number of sites appears to increase, characterized by several localities with thick and rich occupation sequences, such as Umhlatuzana [187] or Sibudu [17, 21, 38, 40]. There are also spatial differences in terms of dominant techno-typological signatures (see [30, 32, 190]). Combining geographical with chronological information, our results from Sibudu and Holley Shelter, as well as other recent studies [23, 30, 31, 179, 182, 185, 191], demonstrate that MSA lithic technology during MIS 3 in southern Africa may well be characterized by an increase in variability and regionalization compared to the previous HP and SB. At least in KwaZulu-Natal, this pattern cannot be explained by demographic collapses or technological regression (*sensu* [26, 27, 178, 192, 193]) and it might in part be a reflection of the higher primary productivity of KwaZulu-Natal compared to more marginal environmental zones of southern Africa (cf. [3, 194]). Sibudu in particular exhibits a long and intense occupation sequence with clear techno-typological markers after the HP, showing that knappers continued to use a highly structured and sophisticated lithic technology. We thus interpret the MIS 3 record as showing the evolution of new and divergent technological trajectories with equal or perhaps still greater complexity as those of the earlier SB and HP of southern Africa.

The fine-grained scale of this analysis also shows that we should not underestimate temporal variation below the level of the techno-complex (see also [23, 58, 195, 196] and [2] in particular). Often, MSA site reports treat techno-complexes as monolithic entities, without discussing potential variability within these units. This is ultimately a question of analytical scale reflected in whether researchers combine various find horizons together because they superficially belong to the same techno-complex, or analysis proceeds by the examination of individual find horizons. Unlike other parts of Africa, many archaeological localities of the MSA in southern Africa do provide the necessary resolution, preservation and find density to conduct analyses on very fine temporal and behavioral scales. In our view, this potential has so far not been fully exploited. However, the questions researchers ask and answer with the archaeological record depend largely on the scale of the analysis [1–3]. In order to explain the nature and tempo of cultural evolution among modern humans in Africa, as well as its causes and consequences, we should embrace all scales of analysis.

Finally, our findings suggest that external factors such as climate and environment should be used more prudently as causal explanations for cultural and behavioral change in the MSA (see also [23, 126, 179, 197]). While we do not want to downplay the importance of adaptive responses to variable environments, modern humans in the Pleistocene were able to vary their behavior independent of changes in the natural surroundings. In such cases, internal causality emerging from the social and cultural dynamics within and between groups play a larger role than previously acknowledged, particularly on a fine temporal and spatial scale. These factors include changes in settlement dynamics and social relations, loss and exchange of cultural information, but also independent innovations along with the complex pathways of their subsequent transmission.

## Supporting Information

S1 Fig Distribution of lithic size classes throughout the studied sequence at Sibudu. WOG1 = oldest layer; BSP = youngest layer.
(TIF)

S2 Fig Percentual abundance of techno-functional tool classes. WOG1 = oldest layer; BSP = youngest layer.
(TIF)

S1 Table Frequencies of retouch debitage (<30 mm) for each assemblage at Sibudu. The samples of small debitage combine size classes 5–10 mm and 10–30 mm.
(DOCX)

S2 Table Three measures of reduction intensity for lithic assemblages at Sibudu. A) Blank to core ratio (after [55]). The higher the ratio is, the more intensely reduced are the assemblage. B) Total core mass relative to total assemblage mass (after [56]). The lower the values are, the more intensely reduced were the cores of this assemblage. C) Average core and flake length or thickness (after [54, 55]) Assemblages showing shorter or thinner flakes and cores are more heavily reduced.
(DOCX)

S3 Table Flaking efficiency by layer for all raw materials and for dolerite only at Sibudu. Higher values indicate higher efficiency of converting a mass of stone into flake edge.
(DOCX)

S4 Table Number of blank types used for the manufacture of tools for the combined assemblages WOG1-BSP. (DOCX)

S5 Table Number (n) and proportion (%) of cortex cover on artifacts made on dolerite per assemblage at Sibudu. (DOCX)

S6 Table Number (n) and proportion (%) of small debitage (<30 mm) by raw materials per assemblage at Sibudu. (DOCX)

S7 Table Summary of the most important diachronic changes in lithic technology within WOG1-BSP, highlighting variation in several main technological domains. Domains include raw material procurement, core reduction and preparation, blank production, tool manufacture and lithic density (cf. [53]: 98–137). The color codes indicate homogeneity in frequency or absence/presence of traits. See S2 Text for a statistical comparison of the groupings.
(DOCX)

S1 Text Statistical comparisons of assemblage groups BM-BSP, SU-POX and WOG1-SP. (DOCX)

S2 Text Summary of paleoenvironmental data from Sibudu. (DOCX)
