# Supplementary figures and images for: Examining the Causes and Consequences of Short-Term Behavioral Change during the Middle Stone Age at Sibudu, South Africa
Source: PLoS One. 2015 Jun 22;10(6):e0130001. doi: 10.1371/journal.pone.0130001 (PMC4476744; doi:10.1371/journal.pone.0130001)

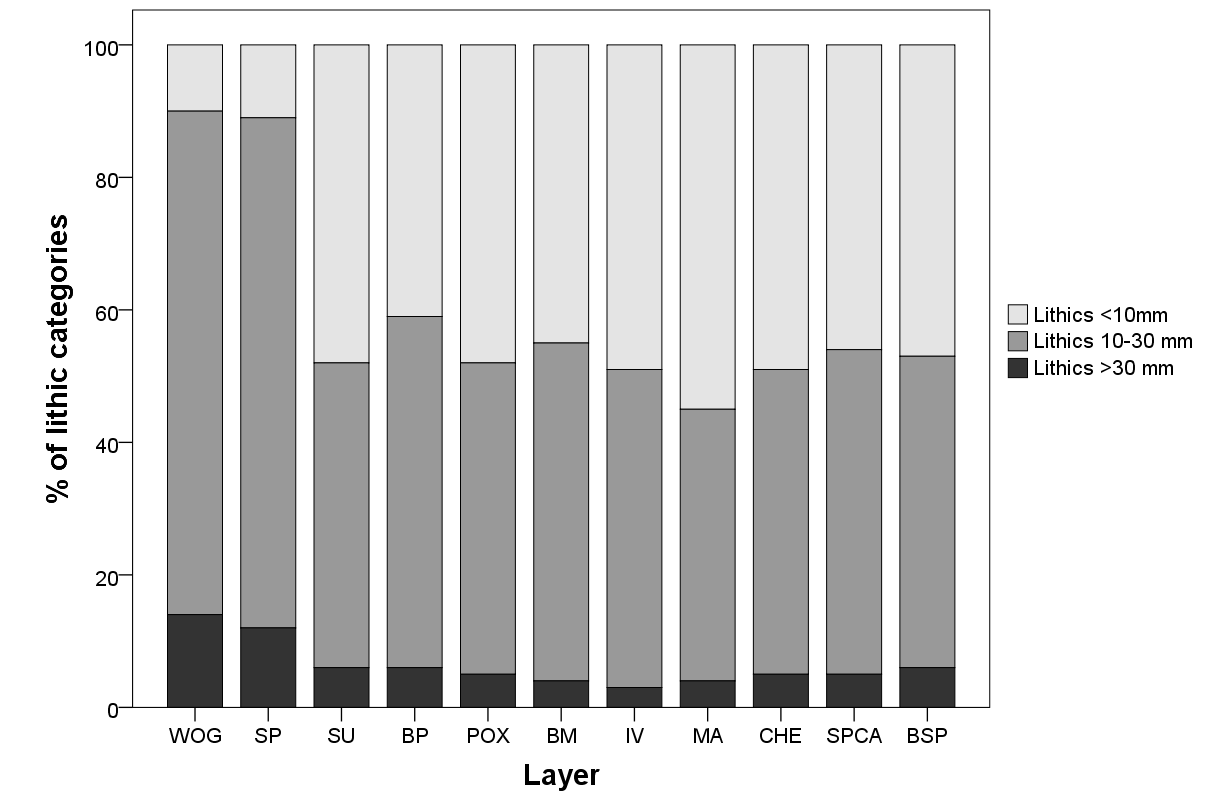

Supplement: S1 Fig — WOG1 = oldest layer; BSP = youngest layer. (TIF) [file pone.0130001.s001.tif]

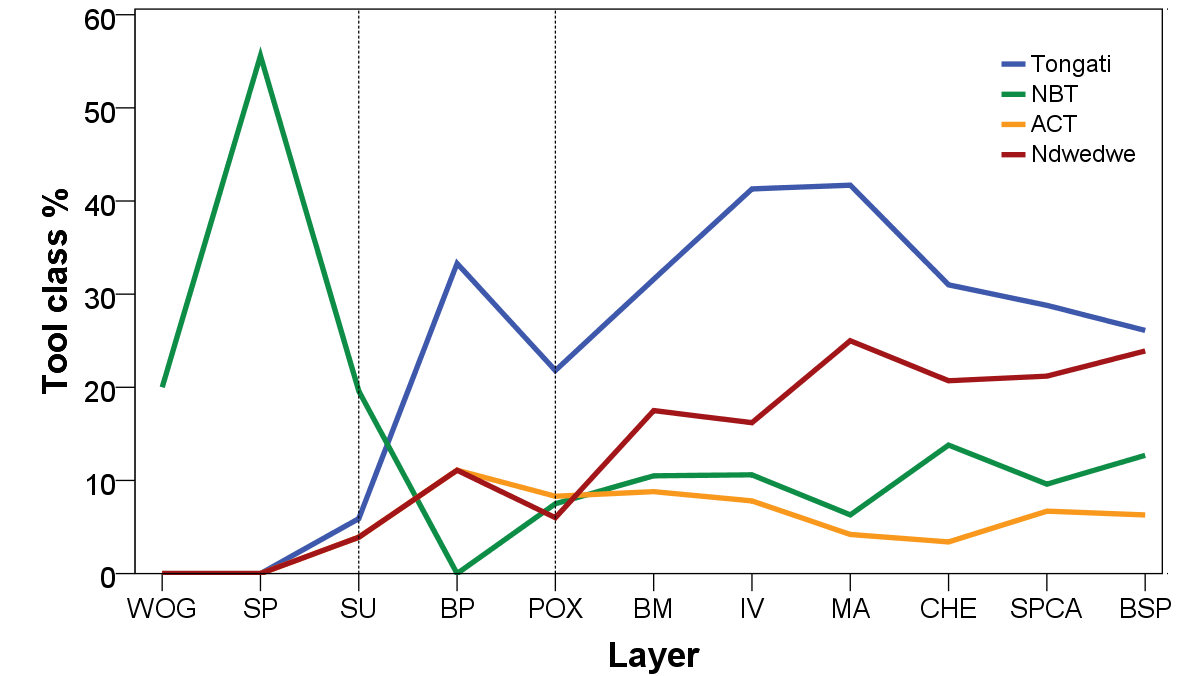

Supplement: S2 Fig — WOG1 = oldest layer; BSP = youngest layer. (TIF) [file pone.0130001.s002.tif]
