# Supplementary material for: Examining the Causes and Consequences of Short-Term Behavioral Change during the Middle Stone Age at Sibudu, South Africa
Source: PLoS One. 2015 Jun 22;10(6):e0130001. doi: 10.1371/journal.pone.0130001 (PMC4476744; doi:10.1371/journal.pone.0130001)
Supplement: S1 Table — The samples of small debitage combine size classes 5–10 mm and 10–30 mm. (DOCX) [file pone.0130001.s003.docx]

**S1 Table. Frequencies of retouch debitage (<30 mm) for each assemblage at Sibudu**. The samples of small debitage combine size classes 5-10 mm and 10-30 mm.

| **Layer** | **Retouch**  **Debitage (n)** | **Debitage**  **Total (n)** | **Retouch**  **Debitage (%)** |
| --- | --- | --- | --- |
| BSP | 303 | 2221 | 13.6 |
| SPCA | 238 | 1019 | 23.4 |
| CHE | 48 | 199 | 24.1 |
| MA | 49 | 231 | 21.2 |
| IV | 526 | 3358 | 15.7 |
| BM | 123 | 1175 | 10.5 |
| POX | 102 | 2883 | 3.5 |
| BP | 117 | 3411 | 3.4 |
| SU | 74 | 3666 | 2.0 |
| SP | 44 | 2603 | 1.7 |
| WOG1 | 21 | 1920 | 1.1 |
| TOTAL | 1645 | 22686 | 7.3 |
