# Supplementary material for: Examining the Causes and Consequences of Short-Term Behavioral Change during the Middle Stone Age at Sibudu, South Africa
Source: PLoS One. 2015 Jun 22;10(6):e0130001. doi: 10.1371/journal.pone.0130001 (PMC4476744; doi:10.1371/journal.pone.0130001)
Supplement: S2 Table — A) Blank to core ratio (after [55]). The higher the ratio is, the more intensely reduced are the assemblage. B) Total core mass relative to total assemblage mass (after [56]). The lower the values are, the more intensely reduced were the cores of this assemblage. C) Average core and flake length or thickness (after [54, 55]) Assemblages showing shorter or thinner flakes and cores are more heavily reduced. (DOCX) [file pone.0130001.s004.docx]

**S2 Table. Three measures of reduction intensity for lithic assemblages at Sibudu.**

A) Blank to core ratio (after [55]). The higher the ratio is, the more intensely reduced are the assemblage.

| **Layer** | **Blanks (n)** | **Cores (n)** | **Blank/Core**  **ratio** |
| --- | --- | --- | --- |
| BSP | 780 | 19 | 41.1 |
| SPCA | 557 | 12 | 46.4 |
| CHE | 128 | 3 | 42.7 |
| MA | 171 | 1 | 171.0 |
| IV | 652 | 14 | 46.6 |
| BM | 253 | 3 | 84.3 |
| POX | 2151 | 12 | 179.3 |
| BP | 259 | 1 | 259.0 |
| SU | 1591 | 11 | 144.6 |
| SP | 689 | 6 | 114.8 |
| WOG1 | 357 | 5 | 71.4 |
| Total | 7588 | 87 | 87.2 |

B) Total core mass relative to total assemblage mass (after [56]). The lower the values are, the more intensely reduced were the cores of this assemblage.

| **Layer** | **Core mass (g)** | **Blank mass (g)** | **Ratio** |
| --- | --- | --- | --- |
| BSP | 961 | 11860 | 0.081 |
| SPCA | 362 | 7870 | 0.046 |
| CHE | 95 | 1499 | 0.063 |
| MA | 34 | 1950 | 0.017 |
| IV | 580 | 6988 | 0.083 |
| BM | 175 | 2196 | 0.080 |
| POX | 346 | 16759 | 0.021 |
| BP | 37 | 2194 | 0.017 |
| SU | 310 | 13619 | 0.023 |
| SP | 189 | 6160 | 0.031 |
| WOG1 | 39^1^ | 3563 | 0.011 |
| Total | 3128 | 74658 | 0.042 |

^1^ This values is highly influenced by the majority of cores being of quartz.

C) Average core and flake length or thickness (after [54, 55]) Assemblages showing shorter or thinner flakes and cores are more heavily reduced.

| **Layer** | **Blank MD^1^ (mm)** | **Blank thickness (mm)** | **Core MD^1^ (mm)** | **Core thickness (mm)** |
| --- | --- | --- | --- | --- |
| BSP | 48.2 | 8.4 | 49.2 | 20.2 |
| SPCA | 45.0 | 8.7 | 44.8 | 17.1 |
| CHE | 42.0 | 8.5 | 50.7 | 19.5 |
| MA | 46.9 | 9.1 | 47.0 | 16.0 |
| IV | 42.1 | 8.1 | 48.6 | 19.4 |
| BM | 40.6 | 7.8 | 51.0 | 22.3 |
| POX | 40.2 | 7.5 | 45.2 | 18.7 |
| BP | 41.5 | 7.6 | 46.0 | 20.0 |
| SU | 40.2 | 8 | 46.5 | 17.2 |
| SP | 39.8 | 8.4 | 45.3 | 16.5 |
| WOG1 | 42.2 | 9.1 | 26.4^2^ | 12.8^2^ |
| Total | 41.8 | 8.1 | 46.1 | 18.3 |

^1^ Maximum dimension

^2^ These values are highly influenced by the majority of cores being of quartz.
