# Supplementary material for: Examining the Causes and Consequences of Short-Term Behavioral Change during the Middle Stone Age at Sibudu, South Africa
Source: PLoS One. 2015 Jun 22;10(6):e0130001. doi: 10.1371/journal.pone.0130001 (PMC4476744; doi:10.1371/journal.pone.0130001)
Supplement: S3 Table — Higher values indicate higher efficiency of converting a mass of stone into flake edge. (DOCX) [file pone.0130001.s005.docx]

**S3** **Table. Flaking efficiency by layer for all raw materials and for dolerite only at Sibudu**. Higher values indicate higher efficiency of converting a mass of stone into flake edge.

| **Layer** | **n (all)** | **Flaking**  **efficiency Ø^1^** | **n (dolerite)** | **Flaking**  **efficiency Ø^1^** |
| --- | --- | --- | --- | --- |
| BSP | 247 | 13.1 | 163 | 11.0 |
| SPCA | 187 | 14.1 | 103 | 12.7 |
| CHE | 35 | 15.9 | 21 | 13.7 |
| MA | 43 | 13.3 | 25 | 14.2 |
| IV | 188 | 17.5 | 119 | 14.4 |
| BM | 77 | 17.1 | 60 | 15.9 |
| POX | 570 | 18.7 | 517 | 18.2 |
| BP | 95 | 17.5 | 86 | 17.3 |
| SU | 704 | 17.1 | 656 | 17.0 |
| SP | 341 | 16.3 | 291 | 16.4 |
| WOG1 | 150 | 14.2 | 130 | 14.3 |
| Total | 2637 | 16.6 | 2171 | 16.1 |

^1^Flaking efficiency is calculated following Mackay [58].
