# Supplementary material for: Examining the Causes and Consequences of Short-Term Behavioral Change during the Middle Stone Age at Sibudu, South Africa
Source: PLoS One. 2015 Jun 22;10(6):e0130001. doi: 10.1371/journal.pone.0130001 (PMC4476744; doi:10.1371/journal.pone.0130001)
Supplement: S4 Table — (DOCX) [file pone.0130001.s006.docx]

**Table S4. Number of blank types used for the manufacture of tools for the combined assemblages WOG1-BSP.**

| **Blank** | **Tools (n)** | **Tools (%)^1^** | **Blanks (%)^2^** | **%diff^3^** | **BRI^4^** |
| --- | --- | --- | --- | --- | --- |
| Flake | 378 | 49.7 | 74.4 | -24.7 | 0.67 |
| Convergent Flake | 242 | 31.8 | 11.8 | +20.0 | 2.69 |
| Blade | 136 | 17.9 | 13.2 | +4.7 | 1.36 |
| Bladelet | 4 | 0.5 | 0.6 | -0.1 | 0.83 |

^1^Proportion of tools made on this blank type in all assemblages

^2^Proportion of this blank type among all blanks in all assemblages.

^3^Tools (%) – Blanks (%)

^4^BRI = Blank Retouch Index: Tools (%) / Blanks (%)
