# Supplementary material for: Examining the Causes and Consequences of Short-Term Behavioral Change during the Middle Stone Age at Sibudu, South Africa
Source: PLoS One. 2015 Jun 22;10(6):e0130001. doi: 10.1371/journal.pone.0130001 (PMC4476744; doi:10.1371/journal.pone.0130001)
Supplement: S5 Table — (DOCX) [file pone.0130001.s007.docx]

**S5 Table. Number (n) and proportion (%) of cortex cover on artifacts made on dolerite per assemblage at Sibudu.**

|  | **BSP** | **SPCA** | **CHE** | **MA** | **IV** | **BM** | **POX** | **BP** | **SU** | **SP** | **WOG1** |
| --- | --- | --- | --- | --- | --- | --- | --- | --- | --- | --- | --- |
| 0% | 322 | 199 | 35 | 65 | 221 | 119 | 1333 | 174 | 1091 | 394 | 217 |
| 1-20% | 83 | 38 | 16 | 15 | 60 | 20 | 255 | 24 | 147 | 70 | 12 |
| 21-40% | 63 | 43 | 14 | 11 | 52 | 16 | 143 | 18 | 129 | 40 | 22 |
| 41-60% | 21 | 16 | 4 | 7 | 30 | 14 | 66 | 9 | 39 | 18 | 3 |
| 61-80% | 28 | 24 | 3 | 8 | 28 | 8 | 76 | 13 | 48 | 29 | 8 |
| 81-99% | 13 | 8 | 5 | 2 | 12 | 2 | 30 | 5 | 15 | 14 | 4 |
| 100% | 4 | 5 | 2 | 3 | 1 | 2 | 12 | 2 | 9 | 1 | 4 |
| Total | 534 | 333 | 79 | 111 | 404 | 181 | 1333 | 245 | 1478 | 566 | 270 |

All values are numbers (n)

|  | **BSP** | **SPCA** | **CHE** | **MA** | **IV** | **BM** | **POX** | **BP** | **SU** | **SP** | **WOG1** |
| --- | --- | --- | --- | --- | --- | --- | --- | --- | --- | --- | --- |
| 0% | 60.3 | 59.8 | 44.3 | 58.6 | 54.7 | 65.7 | 69.6 | 71.0 | 73.8 | 69.6 | 80.4 |
| 1-20% | 15.5 | 11.4 | 20.3 | 13.5 | 14.9 | 11.0 | 13.3 | 9.8 | 9.9 | 12.4 | 4.4 |
| 21-40% | 11.8 | 12.9 | 17.7 | 9.9 | 12.9 | 8.8 | 7.5 | 7.3 | 8.7 | 7.1 | 8.1 |
| 41-60% | 3.9 | 4.8 | 5.1 | 6.3 | 7.4 | 7.7 | 3.4 | 3.7 | 2.6 | 3.2 | 1.1 |
| 61-80% | 5.2 | 7.2 | 3.8 | 7.2 | 6.9 | 4.4 | 4.0 | 5.3 | 3.2 | 5.1 | 3.0 |
| 81-99% | 2.4 | 2.4 | 6.3 | 1.8 | 3.0 | 1.1 | 1.6 | 2.0 | 1.0 | 2.5 | 1.5 |
| 100% | 0.7 | 1.5 | 2.5 | 2.7 | 0.2 | 1.1 | 0.6 | 0.8 | 0.6 | 0.2 | 1.5 |

All values are percentages (%).
