# Supplementary material for: Examining the Causes and Consequences of Short-Term Behavioral Change during the Middle Stone Age at Sibudu, South Africa
Source: PLoS One. 2015 Jun 22;10(6):e0130001. doi: 10.1371/journal.pone.0130001 (PMC4476744; doi:10.1371/journal.pone.0130001)
Supplement: S6 Table — (DOCX) [file pone.0130001.s008.docx]

**S6 Table. Number (n) and proportion (%) of small debitage (<30 mm) by raw materials per assemblage at Sibudu.**

a) Size class 10-30 mm:

| **Layer** | **Dolerite** | **Hornfels** | **Quartzite** | **Quartz** | **Sandstone** | **Other** | **Total** |
| --- | --- | --- | --- | --- | --- | --- | --- |
| SPCA | 470 | 517 | 7 | 4 | 10 | 1 | 1009 |
| IV | 594 | 469 | 3 | 1 | 24 | 11 | 1102 |
| BP | 1697 | 91 | 18 | 0 | 116 | 0 | 1922 |
| SU | 1604 | 17 | 38 | 2 | 158 | 8 | 1827 |
| SP | 1743 | 5 | 44 | 4 | 304 | 3 | 2103 |
| WOG1 | 1097 | 2 | 53 | 68 | 490 | 2 | 1712 |
| Total | 7205 | 1101 | 163 | 79 | 1102 | 25 | 9675 |

All values are numbers (n).

| **Layer** | **Dolerite** | **Hornfels** | **Quartzite** | **Quartz** | **Sandstone** | **Other** |
| --- | --- | --- | --- | --- | --- | --- |
| SPCA | 46.6 | 51.2 | 0.7 | 0.4 | 1.0 | 0.1 |
| IV | 53.9 | 42.6 | 0.3 | 0.1 | 2.2 | 1.0 |
| BP | 88.3 | 4.7 | 0.9 | 0.0 | 6.0 | 0.0 |
| SU | 87.8 | 0.9 | 2.1 | 0.1 | 8.6 | 0.4 |
| SP | 82.9 | 0.2 | 2.1 | 0.2 | 14.5 | 0.1 |
| WOG1 | 64.1 | 0.1 | 3.1 | 4.0 | 28.6 | 0.1 |
| Total | 74.5 | 11.4 | 1.7 | 0.8 | 11.4 | 0.3 |

All values are percentages (%).

b) Size class 5-10 mm:

| **Layer** | **Dolerite** | **Hornfels** | **Quartzite** | **Quartz** | **Sandstone** | **Other** | **Total** |
| --- | --- | --- | --- | --- | --- | --- | --- |
| SPCA | 409 | 447 | 3 | 4 | 10 | 2 | 875 |
| IV | 568 | 430 | 4 | 2 | 11 | 5 | 1020 |
| BP | 1294 | 94 | 15 | 4 | 80 | 2 | 1489 |
| SU | 1623 | 23 | 43 | 4 | 143 | 3 | 1839 |
| SP | 415 | 2 | 9 | 9 | 62 | 2 | 499 |
| WOG1 | 131 | 0 | 7 | 14 | 56 | 0 | 208 |
| Total | 4440 | 996 | 81 | 37 | 362 | 14 | 5930 |

All values are numbers (n).

| **Layer** | **Dolerite** | **Hornfels** | **Quartzite** | **Quartz** | **Sandstone** | **Other** |
| --- | --- | --- | --- | --- | --- | --- |
| SPCA | 46.7 | 51.1 | 0.3 | 0.5 | 1.1 | 0.2 |
| IV | 55.7 | 42.2 | 0.4 | 0.2 | 1.1 | 0.5 |
| BP | 86.9 | 6.3 | 1.0 | 0.3 | 5.4 | 0.1 |
| SU | 88.3 | 1.3 | 2.3 | 0.2 | 7.8 | 0.2 |
| SP | 83.2 | 0.4 | 1.8 | 1.8 | 12.4 | 0.4 |
| WOG1 | 63.0 | 0.0 | 3.4 | 6.7 | 26.9 | 0.0 |
| Total | 74.9 | 16.8 | 1.4 | 0.6 | 6.1 | 0.2 |

All values are percentages (%).
