# Supplementary material for: Examining the Causes and Consequences of Short-Term Behavioral Change during the Middle Stone Age at Sibudu, South Africa
Source: PLoS One. 2015 Jun 22;10(6):e0130001. doi: 10.1371/journal.pone.0130001 (PMC4476744; doi:10.1371/journal.pone.0130001)
Supplement: S7 Table — Domains include raw material procurement, core reduction and preparation, blank production, tool manufacture and lithic density (cf. [53]: 98–137). The color codes indicate homogeneity in frequency or absence/presence of traits. See S2 Text for a statistical comparison of the groupings. (DOCX) [file pone.0130001.s009.docx]

**S7 Table.** **Summary of the most important diachronic changes in lithic technology within WOG1-BSP, highlighting variation in several main technological domains.** Domains include raw material procurement, core reduction and preparation, blank production, tool manufacture and lithic density (*cf*. [53]: 98-137). The color codes indicate homogeneity in frequency or absence/presence of traits. See S1 Text for a statistical comparison of the groupings.

| **Layer** | **RMU non-**  **local (%)** | **RMU**  **selection** | **Core**  **reduction** | **Blank**  **production** | **Facet. Platf. (%)** | **Tool**  **%** | **Tool classes (n)** | **Tong. & Ndw. (%)** | **Unifac. Points / notched (n)** | **Lithic density (n/m³)** |
| --- | --- | --- | --- | --- | --- | --- | --- | --- | --- | --- |
| **BSP** | 32 | 1) Dolerite  2) Hornfels  3) Sandstone | 1) Platform laminar  2) Parallel/Levallois | 1) Flake  2) Blade  3) Point | 27 | 17 | 4/4 | 50 | 67 / 4 | 37.400 |
| **SPCA** | 38 | 1) Dolerite  2) Hornfels  3) Sandstone | 1) Platform laminar  2) Parallel/Levallois | 1) Flake  2) Blade  3) Point | 23 | 18 | 4/4 | 50 | 48 / 4 | 35.300 |
| **CHE** | 38 | 1) Dolerite  2) Hornfels  3) Sandstone | 1) Platform laminar  2) Parallel/Levallois | 1) Flake  2) Blade  3) Point | 25 | 22 | 4/4 | 52 | 11 / 0 | 32.500 |
| **MA** | 33 | 1) Dolerite  2) Hornfels  3) Sandstone | 1) Platform laminar  2) Parallel/Levallois | 1) Flake  2) Point  3) Blade | 22 | 27 | 4/4 | 67 | 26 / 0 | 33.800 |
| **IV** | 35 | 1) Dolerite  2) Hornfels  3) Sandstone | 1) Platform laminar  2) Parallel/Levallois | 1) Flake  2) Blade  3) Point | 25 | 27 | 4/4 | 58 | 97 / 11 | 49.700 |
| **BM** | 25 | 1) Dolerite  2) Hornfels  3) Sandstone | 1) Platform laminar  2) Parallel/Levallois | 1) Flake  2) Blade  3) Point | 29 | 22 | 4/4 | 49 | 29 / 0 | 40.400 |
| **POX** | 6 | **1) Dolerite**  2) Hornfels  3) Sandstone | 1) Inclined/Discoid  2) Platform laminar | 1) Flake  2) Blade  3) Point | 16 | 6 | 4/4 | 28 | 49 / 17 | 79.400 |
| **BP** | 3 | **1) Dolerite**  2) Hornfels  3) Sandstone | 1) Inclined/Discoid  2) Platform laminar | 1) Flake  2) Point  3) Blade | 15 | 3 | 3/4 | 44 | 5 / 0 | 89.600 |
| **SU** | 1 | **1) Dolerite**  2) Sandstone  3) Quartzite | 1) Inclined/Discoid  2) Platform laminar | 1) Flake  2) Blade  3) Point | 12 | 3 | 4/4 | 10 | 14 / 10 | 71.200 |
| **SP** | 1 | 1) Dolerite  2) Sandstone  3) Quartzite | 1) Parallel/Levallois  2) Inclined/Discoid | 1) Flake  2) Point  3) Blade | 23 | 1 | 1/4 | 0 | 0 / 7 | 24.900 |
| **WOG1** | 0 | 1) Dolerite  2) Sandstone  3) Quartzite | 1) Parallel/Levallois  2) Inclined/Discoid | 1) Flake  2) Point  3) Blade | 23 | 1 | 1/4 | 0 | 1 / 2 | 13.700 |
| **Layer** | **RMU non-**  **local (%)** | **RMU**  **selection** | **Core**  **reduction** | **Blank**  **production** | **Facet. Platf. (%)** | **Tool**  **%** | **Tool classes (n)** | **Tong. & Ndw. (%)** | **Unifac. Points / notched (n)** | **Lithic density (n/m³)** |
| **BSP**  **SPCA**  **CHE**  **MA**  **IV**  **BM** | High  (25-38%) | 1) Dolerite  2) Hornfels  *very frequent*  3) Sandstone | Platform laminar dominates,  Parallel/Levallois common | 1) Flake  2) Blade  3) Point | High  (22-29%) | High  (17-27%) | All present | High  (49-67%) | Unifacial points very frequent, Notches rare or absent | Intermediate  (33.000-50.000) |
| **POX**  **BP**  **SU** | Very low  (0-6%) | 1) Dolerite  dominance  2) Hornfels  3) Sandstone | Inclined/Discoid dominates,  Platform laminar common |  | Intermediate  (12-16%) | Very low  (1-6%) |  | Inter-mediate  (10-44%) | Unifacial points frequent, notches common | Very high  (81.000-90.000) |
| **SP**  **WOG1** |  | 1) Dolerite  2) Sandstone  3) Quartzite | Parallel/Levallois dominates, Inclined/Discoid common | 1) Flake  2) Point | High  (23%) |  | Only NBTs | Absent  (0%) | More notches than unifacial points | Low  (14.000-25.000) |
