# Supplementary material for: Examining the Causes and Consequences of Short-Term Behavioral Change during the Middle Stone Age at Sibudu, South Africa
Source: PLoS One. 2015 Jun 22;10(6):e0130001. doi: 10.1371/journal.pone.0130001 (PMC4476744; doi:10.1371/journal.pone.0130001)
Supplement: S1 Text — (DOCX) [file pone.0130001.s010.docx]

**S1 Text. Statistical comparisons of assemblage groups BM-BSP, SU-POX and WOG1-SP**

For a statistical analyses of differences between the three discerned assemblage groups of the Sibudu sequence (BM-BSP; SU-POX; WOG1-SP), chi-square tests for homogeneity were performed. The tests determine whether frequency counts of a categorical variable are distributed identically across different populations (*i.e.* assemblage groups). The main domains analyzed were raw material selection, debitage distribution, blank production, core reduction and tool manufacture (*cf*. [53]: 98-137). Numerical data of individual assemblages were combined for each of the three groups and then compared via chi square tests. All 3x3, 2x3 and 2x2 chi square tests were performed with Yates’ correction. For some of the comparisons between two groups (2x2), Fisher’s exact test was employed due to low sample sizes (marked with asterisks). All tests were two-tailed with α=0.05 and calculated in SPSS 19.0 for Windows. Statistically significant results (*p*<0.05) indicate that the distributions of traits are not homogeneous for the three (respectively two) groupings.

**1) Raw material distribution**

**Test 1**

|  | **Dolerite** | **Hornfels** | **Sandstone** | **Total χ² (df=4)** | **1 vs. 2 (df=2)** | **1 vs. 3 (df=2)** | **2 vs. 3 (df=2)** |
| --- | --- | --- | --- | --- | --- | --- | --- |
| BM-BSP (1) | 1645 | 893 | 63 | 1595.598; p<0.001 | 1034.818;  p<0.001 | 608.03;  p<0.001 | 216.085;  p<0.001 |
| SU-POX (2) | 3643 | 163 | 178 |  |  |  |  |
| WOG1-SP (3) | 837 | 7 | 174 |  |  |  |  |

**Test 2**

|  | **Dolerite** | **Non-Dolerite** | **Total χ² (df=2)** | **1 vs. 2 (df=1)** | **1 vs. 3 (df=1)** | **2 vs. 3 (df=1)** |
| --- | --- | --- | --- | --- | --- | --- |
| BM-BSP (1) | 1645 | 1004 | 706.192; p<0.001 | 708.045; p<0.001 | 86.26; p<0.001 | 95.679; p<0.001 |
| SU-POX (2) | 3643 | 434 |  |  |  |  |
| WOG1-SP (3) | 837 | 236 |  |  |  |  |

**Test 3**

|  | **Local** | **Non-local** | **Total χ² (df=2)** | **1 vs. 2 (df=1)** | **1 vs. 3 (df=1)** | **2 vs. 3 (df=1)** |
| --- | --- | --- | --- | --- | --- | --- |
| BM-BSP (1) | 1756 | 893 | 1378.564; p<0.001 | 1068.831; p<0.001 | 453.421; p<0.001 | 28.794; p<0.001 |
| SU-POX (2) | 3914 | 163 |  |  |  |  |
| WOG1-SP (3) | 1066 | 7 |  |  |  |  |

Interpretation: The chi-square tests for homogeneity show that the distribution of raw materials between the three groups is statistically different (p<0.001) at all levels of comparison.

**2) Debitage category distribution**

**Test 1**

|  | **Blank** | **Tool** | **Core** | **Total χ² (df=4)** | **1 vs. 2 (df=2)** | **1 vs. 3 (df=2)** | **2 vs. 3 (df=2)** |
| --- | --- | --- | --- | --- | --- | --- | --- |
| BM-BSP (1) | 1981 | 559 | 52 | 620.782; p<0.001 | 468.912; p<0.001 | 238.777; p<0.001 | 27.668; p<0.001 |
| SU-POX (2) | 3808 | 198 | 24 |  |  |  |  |
| WOG1-SP (3) | 1032 | 14 | 11 |  |  |  |  |

**Test 2**

|  | **Tool** | **Non-tool** | **Total χ² (df=2)** | **1 vs. 2 (df=1)** | **1 vs. 3 (df=1)** | **2 vs. 3 (df=1)** |
| --- | --- | --- | --- | --- | --- | --- |
| BM-BSP (1) | 559 | 2090 | 575.764; p<0.001 | 422.666; p<0.001 | 228.284; p<0.001 | 26.256; p<0.001 |
| SU-POX (2) | 198 | 3879 |  |  |  |  |
| WOG1-SP (3) | 14 | 1059 |  |  |  |  |

Interpretation: The chi-square tests for homogeneity show that the distribution of debitage categories between the three groups is statistically different (p<0.001) at all levels of comparison.

**3) Blank type distribution**

**Test 1**

|  | **Blade** | **Flake** | **Point** | **Total χ² (df=4)** | **1 vs. 2 (df=2)** | **1 vs. 3 (df=2)** | **2 vs. 3 (df=2)** |
| --- | --- | --- | --- | --- | --- | --- | --- |
| BM-BSP (1) | 393 | 1802 | 332 | 93.983;  p<0.001 | 43.654; p<0.001 | 75.975; p<0.001 | 26.432; p<0.001 |
| SU-POX (2) | 399 | 2976 | 436 |  |  |  |  |
| WOG1-SP (3) | 54 | 863 | 126 |  |  |  |  |

**Test 2**

|  | **Blade** | **Flake** | **Total χ² (df=2)** | **1 vs. 2 (df=1)** | **1 vs. 3 (df=1)** | **2 vs. 3 (df=1)** |
| --- | --- | --- | --- | --- | --- | --- |
| BM-BSP (1) | 393 | 1802 | 90.399;  p<0.001 | 39.84; p<0.001 | 74.941; p<0.001 | 26.266; p<0.001 |
| SU-POX (2) | 399 | 2976 |  |  |  |  |
| WOG1-SP (3) | 54 | 863 |  |  |  |  |

Interpretation: The chi-square tests for homogeneity show that the distribution of blank types between the three groups is statistically different (p<0.001) at all levels of comparison.

**4) Core type distribution**

**Test 1**

|  | **Platform** | **Parallel** | **Inclined** | **Total χ² (df=4)** | **1 vs. 2 (df=2)** | **1 vs. 3 (df=2)** | **2 vs. 3 (df=2)** |
| --- | --- | --- | --- | --- | --- | --- | --- |
| BM-BSP (1) | 19 | 23 | 3 | - ^1^ | 3.543; p=0.17 | - ^1^ | - ^1^ |
| SU-POX (2) | 7 | 6 | 5 |  |  |  |  |
| WOG1-SP (3) | 3 | 4 | 0 |  |  |  |  |

^1^ At least one expected frequency is <1.

**Test 2**

|  | **Platform** | **Non-platform** | **Total χ² (df=2)** | **1 vs. 2 (df=1)** | **1 vs. 3 (df=1)** | **2 vs. 3 (df=1)** |
| --- | --- | --- | --- | --- | --- | --- |
| BM-BSP (1) | 19 | 33 | 0.176;  p=0.916 | 0.137; p=0.711 | 0.056; p=0.813 | 0.083; p=0.773 |
| SU-POX (2) | 7 | 17 |  |  |  |  |
| WOG1-SP (3) | 3 | 8 |  |  |  |  |

**Test 3**

|  | **Parallel** | **Non-parallel** | **Total χ² (df=2)** | **1 vs. 2 (df=1)** | **1 vs. 3 (df=1)** | **2 vs. 3 (df=1)** |
| --- | --- | --- | --- | --- | --- | --- |
| BM-BSP (1) | 23 | 29 | 1.87;  p=0.393 | 1.823; p=0.177 | 0.021; p=0.884 | 0.083; p=0.773 |
| SU-POX (2) | 6 | 18 |  |  |  |  |
| WOG1-SP (3) | 4 | 7 |  |  |  |  |

Interpretation: The chi-square tests for homogeneity show that the distribution of core types between the three groups is not statistically different (p>0.17), indicating a homogeneous distribution. This pattern is most likely the result of the scarcity of cores and their intense degree of exploitation (see *Reduction of cores* in main text)

**5) Tool type distribution**

**Test 1**

|  | **Unifacial point** | **Notch & Denticulate** | **Side scraper** | **Total χ² (df=4)** | **1 vs. 2 (df=2)** | **1 vs. 3 (df=2)** | **2 vs. 3 (df=2)** |
| --- | --- | --- | --- | --- | --- | --- | --- |
| BM-BSP (1) | 278 | 19 | 95 | 82.168; p<0.001 | 34.068; p<0.001 | - ^1^ | 14.081; p<0.001 |
| SU-POX (2) | 68 | 27 | 22 |  |  |  |  |
| WOG1-SP (3) | 1 | 9 | 1 |  |  |  |  |

^1^ At least one expected frequency is <1.

**Test 2**

|  | **Unifacial point** | **Notch & Denticulate** | **Total χ² (df=2)** | **1 vs. 2 (df=1)** | **1 vs. 3 (df=1)** | **2 vs. 3 (df=1)** |
| --- | --- | --- | --- | --- | --- | --- |
| BM-BSP (1) | 278 | 19 | 72.054; p<0.001 | 31.614; p<0.001 | p<0.001* | p<0.001* |
| SU-POX (2) | 68 | 27 |  |  |  |  |
| WOG1-SP (3) | 1 | 9 |  |  |  |  |

**Test 3**

|  | **Unifacial point** | **Rest of tools** | **Total χ² (df=2)** | **1 vs. 2 (df=1)** | **1 vs. 3 (df=1)** | **2 vs. 3 (df=1)** |
| --- | --- | --- | --- | --- | --- | --- |
| BM-BSP (1) | 278 | 281 | 20.175; p<0.001 | 13.339; p<0.001 | 8.284; p=0.004 | 3.255;  p=0.07 |
| SU-POX (2) | 68 | 130 |  |  |  |  |
| WOG1-SP (3) | 1 | 13 |  |  |  |  |

Interpretation: The chi-square tests for homogeneity show that the distribution of tool types between the three groups is statistically different (p<0.005) at all levels of comparison except one case (p=0.07).

**6) Techno-functional tool class distribution**

**Test 1**

|  | **Tongati** | **Ndwedwe** | **NBT** | **Total χ² (df=4)** | **1 vs. 2 (df=2)** | **1 vs. 3 (df=2)** | **2 vs. 3 (df=2)** |
| --- | --- | --- | --- | --- | --- | --- | --- |
| BM-BSP (1) | 188 | 113 | 60 | 27.200;  p<0.001 | 8.496;  p=0.014 | 21.650;  p<0.001 | - ^1^ |
| SU-POX (2) | 35 | 11 | 20 |  |  |  |  |
| WOG1-SP (3) | 0 | 0 | 6 |  |  |  |  |

^1^ At least one expected frequency is <1.

**Test 2**

|  | **Sibudan tool class** | **Non-Sibudan**  **tool class** | **Total χ² (df=2)** | **1 vs. 2 (df=1)** | **1 vs. 3 (df=1)** | **2 vs. 3 (df=1)** |
| --- | --- | --- | --- | --- | --- | --- |
| BM-BSP (1) | 399 | 240 | 29.714; p<0.001 | 29.097; p<0.001 | 1.477; p=0.22 | 0.01;  p=0.92 |
| SU-POX (2) | 80 | 118 |  |  |  |  |
| WOG1-SP (3) | 6 | 8 |  |  |  |  |

**Test 3**

|  | **Tongati +**  **Ndwedwe + ACT** | **Other** | **Total χ² (df=2)** | **1 vs. 2 (df=1)** | **1 vs. 3 (df=1)** | **2 vs. 3 (df=1)** |
| --- | --- | --- | --- | --- | --- | --- |
| BM-BSP (1) | 339 | 220 | 65.99; p<0.001 | 52.787; p<0.001 | 18.356; p<0.01 | p=0.012* |
| SU-POX (2) | 60 | 138 |  |  |  |  |
| WOG1-SP (3) | 0 | 14 |  |  |  |  |

Interpretation: The chi-square tests for homogeneity show that the distribution of techno-functional tool classes between the three groups is statistically different (p<0.014) in 9 of 11 comparisons.
