# Supplementary material for: Examining the Causes and Consequences of Short-Term Behavioral Change during the Middle Stone Age at Sibudu, South Africa
Source: PLoS One. 2015 Jun 22;10(6):e0130001. doi: 10.1371/journal.pone.0130001 (PMC4476744; doi:10.1371/journal.pone.0130001)
Supplement: S2 Text — (DOCX) [file pone.0130001.s011.docx]

**S2 Text. Summary of paleoenvironmental data from Sibudu**

A multitude of studies have provided proxies of paleoenvironments in the archaeological sediments dated to ~58 ka at Sibudu. The table below summarizes the results of these studies pertaining to the sequence WOG1-BSP, listing the proxies used, the interpretation for general environmental trends and the scale of analyses. The scale of the data varies from encompassing the entire sequence (*i.e.* the “post-HP” sediments dating to ~58 ka) to resolution on the level of several or individual archaeological layers. While there are differences between the proxies and minor fluctuations within the sequence, the overall environmental trends are similar for all layers. Other studies that integrated several paleoenvironmental datasets reached corresponding conclusions [17, 126, 158-160].

**Table.** Summary of environmental trends based on various studies at Sibudu.

| **Study [Reference]** | **Environmental trends** | **Proxy** | **Scale / layers** |
| --- | --- | --- | --- |
| Allott 2004  [153] | Climate: Warm and dry  Vegetation: open grassland and some forest vegetation | Charcoal | Layer /  BSP |
| Allott 2006  [155] | Climate: Warm and dry (BSP); cooler (SPCA)  Vegetation: open grassland and some forest vegetation (BSP) / More forest (SPCA) | Charcoal | Layer /  BSP; SPCA |
| Clark 2011  [162] | Vegetation: open grassland and some forest vegetation  (constant) | Macromammals | Sequence /  WOG1-BSP (“post-HP MSA 1”) |
| Clark 2013  [126] | Vegetation: open grassland and some forest vegetation  (constant) | Macromammals | Multi-layer /  WOG1-BSP (post-HP MSA 1 (low) & post-HP MSA 1 (upp)) |
| Clark (personal communication) | Vegetation: open grassland and some forest vegetation  (constant) | Macromammals | Layer /  WOG1-BSP |
| Clark & Plug 2008  [125] | Vegetation: open grassland and some forest vegetation  (constant) | Macromammals | Sequence /  WOG1-BSP (“post-HP MSA 1”) |
| Hall et al. 2014  [160] | Climate: Warm and dry | Charcoal isotopes | Layer / SPCA |
| Herries 2006  [156] | Climate: warm with minor fluctuations to cooler temperatures (but all layers in climatic zone CZ3) | Magnetism | Layer /  WOG1-BSP |
| Renaut & Bamford 2006  [163] | Vegetation. Mix of open grassland and forest vegetation | Pollen | Layer /  CHE; BP |
| Schiegl & Conard 2006  [37] | Climate: Dry with some moisture  Vegetation: Increase in open grassland (BSP hearth)  (constant with minor fluctuations) | Phytoliths & sediment analyses | Layer /  WOG1-BSP |
| Sievers 2006  [164] | Vegetation: Mix of open grassland and forest vegetation (constant with minor fluctuations) | Seeds | Multi-layer /  BSP-BSP2; SPCA-Or; Mi-Su |
| Wadley 2004  [154] | Climate: open grassland and some forest vegetation  (constant) | Seeds | Multi-layer /  SU2-MI; OR-SPCA; BSP-BSP2 |
